# Molecular Rectification: Mechanisms, Modulation Strategies, and Device Realization

**DOI:** 10.1002/advs.78204

**Published:** 2026-10-15

**Authors:** Junnan Guo, Shufan Song, Wenhui Fang, Jifeng Tang, Wenhao Li, Weikang Wu, Shishen Yan, Hui Li, Lishu Zhang

**Affiliations:** ^1^ Key Laboratory for Liquid‐Solid Structural Evolution and Processing of Materials Ministry of Education Shandong University Jinan China; ^2^ School of Physics Shandong University Jinan China

**Keywords:** molecular rectification, molecular electronics, molecular junctions, rectification ratio

## Abstract

Molecular rectifiers, as prototypical components of molecular electronics, present unique opportunities for pushing device miniaturization to its ultimate limits. Nevertheless, challenges including limited rectification ratios, insufficient robustness, and poor reproducibility impede their practical deployment. To make molecular rectifiers competitive with silicon‐based devices, it is important to understand the design principles and fabrication methods from both mechanistic and experimental perspectives. By considering the transport mechanisms, modulation strategies, fabrication and characterization techniques, and theoretical simulations, this review provides a comprehensive overview of molecular rectifiers. Representative examples of conceptually significant and high‐performance molecular rectifier systems are highlighted to illustrate the relationships between rectification mechanisms, molecular design strategies, and device realization. Building on these discussions, we present an outlook for current bottlenecks and future directions to guide the development of molecular rectifiers. This review aims to serve as both a conceptual framework and a technical reference for researchers working at the intersection of molecular electronics and nanoscale device engineering in the post‐CMOS era.

## Graphical abstract

This review focuses on molecular rectification, from its fundamental mechanisms and modulation strategies to device realization. It summarizes the theoretical framework, modulation strategies, fabrication techniques, characterization methods, and modeling and simulation of molecular rectifiers, while discussing challenges and future directions for molecular electronics.

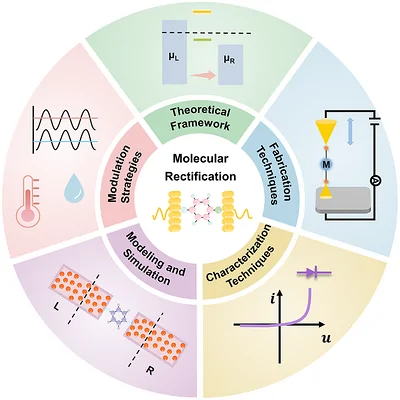

**Abbreviations**
ADF: Amsterdam Density Functional
AFM: Atomic Force Microscopy
ARXPS: Angle‐Resolved X‐ray Photoelectron Spectroscopy
CA: Contact Angle
CV: Cyclic Voltammetry
DFT: Density Functional Theory
DFT–NEGF: Density Functional Theory combined with Non‐Equilibrium Green's Function
DSC: Differential Scanning Calorimetry
EGaIn: Eutectic Gallium–Indium
EPR: Electron Paramagnetic Resonance
ESR: Electron Spin Resonance
FT‐IR: Fourier‐transform Infrared Spectroscopy
HRMS: High‐Resolution Mass Spectrometry
IETS: Inelastic Electron Tunneling Spectroscopy
IRRAS: Infrared Reflection Absorption Spectroscopy
I–t: Current‐Time Measurement
I–V: Current‐Voltage Measurement
I–V–T: Temperature‐dependent Current‐Voltage Measurement
J–V: Current Density‐Voltage Measurement
J–V–T: Current Density‐Voltage‐Temperature Measurement
KPFM: Kelvin Probe Force Microscopy
MCBJ: Mechanically Controllable Break‐Junction
MD: Molecular Dynamics
MOC: Monolayer Organic Crystal
NEXAFS: Near‐Edge X‐ray Absorption Fine Structure
NMR: Nuclear Magnetic Resonance
PL: Photoluminescence
RH: Relative Humidity
RS: Raman Spectroscopy
SAM: Self‐Assembled Monolayer
SEM: Scanning Electron Microscopy
SHG: Second Harmonic Generation
STEM: Scanning Transmission Electron Microscopy
STM: Scanning Tunneling Microscopy
STM‐BJ: Scanning Tunneling Microscope Break‐Junction
TD‐DFT: Time‐Dependent Density Functional Theory
TVS: Transition Voltage Spectroscopy
UPS: Ultraviolet Photoelectron Spectroscopy
UV–vis: Ultraviolet–visible spectroscopy
VASP: Vienna Ab initio Simulation Package
XPBJ: Cross‐plane Break Junction
XPS: X‐ray Photoelectron Spectroscopy
XRD: X‐ray Diffraction

## 1 Introduction

The continuous downscaling of electronic components has pushed conventional silicon‐based technology to the edge of its physical and functional limits [1, 2, 3, 4]. Once device dimensions fall below the sub‐5‐nm node, such systems often exhibit a significant increase in leakage current, degradation of carrier mobility due to short‐channel effects, and enhanced signal interference induced by thermal noise [5, 6, 7, 8, 9, 10]. These non‐ideal physical phenomena affect charge transport, undermining both the performance and scalability of traditional complementary metal–oxide–semiconductor (CMOS) architectures. Against this backdrop, molecular electronics has emerged as a promising alternative by employing single molecules or small ensembles as functional units [11, 12, 13, 14, 15]. Exploiting intrinsic quantum features such as quantum interference and tunable energy levels, molecular electronics not only enables miniaturization toward the sub‐1‐nm regime but also allows the integration of rectification, switching, and memory functionalities within tailored molecular structures [16, 17, 18]. These capabilities provide opportunities for the miniaturization and diversification of post‐Moore electronic systems.

Among various molecular electronic devices, the molecular rectifier stands as both the earliest and the most representative prototype [19, 20, 21]. In 1974, Aviram and Ratner proposed the first theoretical model based on a donor–σ–acceptor (D–σ–A) architecture [12]. By exploiting the energy‐level offset between donor and acceptor units, they theoretically proposed the possibility of unidirectional current flow at the molecular scale. This conceptual mechanism differs from the doping‐dependent rectification of conventional *p*–*n* junctions. Their pioneering proposal established an important conceptual foundation for molecular rectifiers and stimulated subsequent theoretical and experimental studies in molecular electronics. To quantify device performance, the rectification ratio (RR) in this review is defined as the ratio between the larger and smaller absolute currents under opposite biases with the same magnitude. This definition ensures RR ≥ 1, while a higher RR indicates stronger directional selectivity of charge transport and thus greater relevance for practical rectification.

Over the past five decades, both theoretical and experimental studies of molecular rectifiers have advanced significantly, yet their practical device performance remains limited. It was not until 2017 that the first rectifier with an RR of 10^5^ was realized through a self‐assembled monolayer (SAM)‐based junction [22]. Subsequent efforts have pushed RRs into the range of 10^6^–10^8^, but such results are often limited to idealized conditions, falling short of the operational stability and reliability required for practical applications [23, 24]. In most cases, rectification remains modest with RR values below 10^3^ [25, 26, 27]. This limitation can be largely attributed to insufficient asymmetry between molecular orbitals (MOs) and electrode Fermi levels, as well as the difficulty of simultaneously achieving interfacial tunability and long‐term reliability [28, 29, 30]. High operating voltages, limited conductance, and poor scalability further constrain industrial prospects [31, 32, 33].

To overcome these bottlenecks, researchers have advanced the field along three interconnected dimensions: theory, modulation, and experimental realization. Theoretical frameworks have evolved from simple tunneling models to multiscale transport descriptions that incorporate effects such as Fermi‐level pinning and metal‐induced gap states (MIGS) [34, 35, 36, 37]. First‐principles simulations combining density functional theory (DFT) with non‐equilibrium Green's function (NEGF) approaches now provide insights into structure–property relationships and shed light on mechanisms such as quantum interference (QI) and interfacial charge transfer [38, 39, 40]. In parallel, the use of carbon‐based electrodes, including graphene and carbon nanotubes, has contributed to improvements in both interfacial stability and electrical performance. A wide range of external modulation strategies, such as structural engineering and external‐field modulation (e.g., optical, temperature, electrical, and chemical), have opened new avenues toward achieving high‐performance rectifiers [27, 41, 42, 43, 44, 45, 46, 47]. Alongside these advances, device fabrication has progressed from early metal–vacuum–metal tunneling structures to controlled platforms based on mechanically controllable break‐junction (MCBJ) methods [48], scanning tunneling microscope break‐junction (STM‐BJ) methods [49, 50], and SAM‐based junctions [51, 52, 53, 54]. More recently, emerging architectures such as cross‐plane break‐junction (XPBJ) methods, electromigrated nanogap electrodes, and van der Waals (vdW) contacts based on two‐dimensional materials have further improved structural stability and junction reproducibility [55, 56]. In parallel, ordered monolayer strategies, including repeated surface exchange of molecules (ReSEM) and monolayer organic crystals (MOCs), have emerged as effective approaches to improve molecular packing, structural uniformity, and electrical stability, thereby enhancing the reproducibility and robustness of molecular rectifiers [24, 57].

The study of molecular rectifiers has thus evolved from conceptual demonstrations to experimental synthesis and performance optimization [58]. Although previous reviews have provided important summaries of the development of molecular rectifiers, most of them have mainly focused on device classification or conceptual design, while the intrinsic connections among microscopic transport mechanisms, modulation strategies, device fabrication, and characterization remain less systematically discussed [59, 60, 61, 62]. To address this gap, this review is organized along the trajectory from mechanism to modulation and realization, aiming to establish an integrated framework that links molecular‐scale transport mechanisms with device‐level performance. This review focuses primarily on molecular devices in which rectification is governed by individual molecules, molecular assemblies, or molecule–electrode interfaces. It covers both single‐molecule junctions and molecular‐ensemble devices. Accordingly, conventional organic or inorganic diodes are beyond the scope of this review when their rectification behavior is dominated by bulk semiconductor properties and lacks a clearly identified molecular‐scale mechanism. Single‐molecule platforms include dynamic junctions formed using MCBJ, STM‐BJ, and XPBJ methods, as well as static junctions based on carbon electrodes or electromigrated nanogaps, whereas molecular‐ensemble platforms include SAM‐based junctions, molecular multilayers or heterojunctions, and MOCs. The fundamental charge transport principles and classical rectification models are first introduced, followed by a discussion of modulation strategies that involve molecular design, interfacial engineering, quantum effects, and external‐field responses. Advances in fabrication and characterization methods are then surveyed together with developments in first‐principles modeling. To illustrate the evolution of the field, Figure 1 provides a timeline spanning from 1974 to the present, highlighting theoretical milestones and experimental advances. Finally, major challenges and possible future directions are discussed, with an emphasis on the prospective role of molecular rectifiers within the post‐Moore electronic landscape.

**FIGURE 1:**
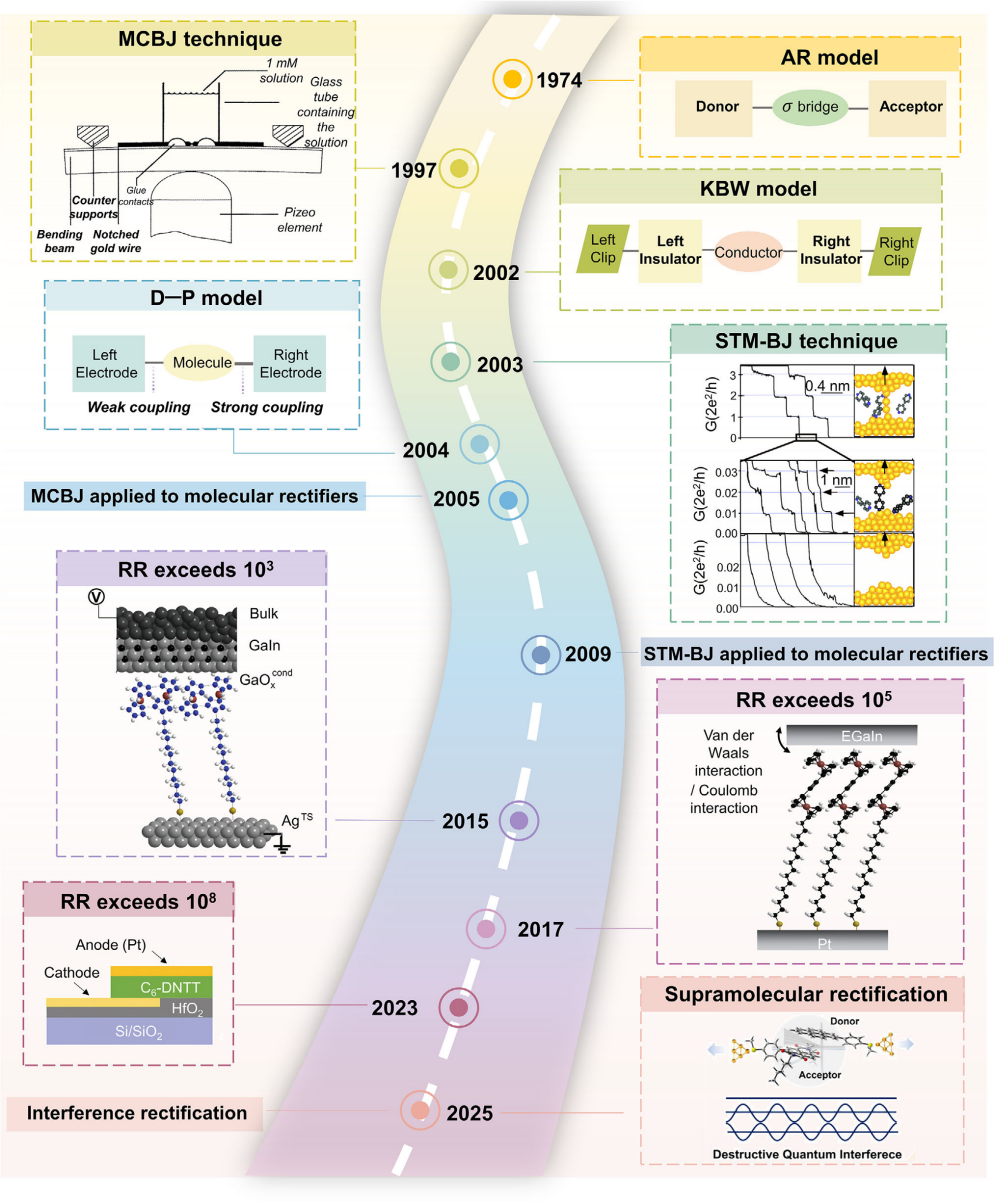
Evolution roadmap and key milestones in the development of molecular rectifiers. The roadmap illustrates the transition from theoretical concepts to experimental realization. Representative advances in rectification ratios (RRs) exceeding 10^3^, 10^5^, and 10^8^ are highlighted, together with recent developments in supramolecular architectures and quantum interference engineering. Reproduced with permission from Ref. [48]. Copyright 1997, The American Association for the Advancement of Science. Reproduced with permission from Ref. [50]. Copyright 2003, The American Association for the Advancement of Science. Reproduced with permission from Ref. [86]. Copyright 2015, American Chemical Society. Reproduced with permission from Ref. [22]. Copyright 2017, Springer Nature. Reproduced from Ref. [24] under the CC BY 4.0 license. Reproduced with permission from Ref. [47]. Copyright 2025, American Chemical Society.

## 2 Theoretical Framework of Molecular Rectification

The mechanisms underlying molecular rectification are complex, arising from the interplay among molecular electronic structure, molecule–electrode interactions, and non‐equilibrium charge transport under applied bias. A thorough understanding of these fundamental physical principles is therefore crucial for the rational design of high‐performance molecular devices with large RRs and reliable operation. This section presents a theoretical framework, from the electronic structure of MOs to the principal rectification mechanisms, laying the foundation for the design of efficient molecular rectifiers.

### 2.1 Molecular Orbitals

Charge transport at the molecular scale is governed by the principles of quantum mechanics [63, 64]. Owing to the small dimensions of molecules, charge carriers are confined within a finite spatial region, resulting in a discrete energy level spectrum [65]. The spacing between adjacent molecular levels is determined primarily by the spatial dimensions of the system and the effective mass of the electrons, and can be qualitatively estimated using the particle‐in‐a‐box model. In general, the smaller the molecular dimensions, the greater the degree of energy level discretization.

These discrete levels, defined as MOs, serve as the fundamental basis for describing the electronic states of a molecule [66]. Their spatial distribution is influenced by the electrostatic potential landscape generated by the nuclei within the molecule. Electron filling of these orbitals follows the Aufbau principle and the Pauli exclusion principle, with each orbital accommodating at most two electrons with opposite spin. By definition, the highest energy MO that is fully occupied by electrons is called the highest occupied molecular orbital (HOMO), which is analogous in physical significance to the valence band maximum in inorganic semiconductors. Conversely, the lowest unoccupied molecular orbital (LUMO) corresponds to the conduction band minimum. As an illustrative example, the energy spectrum of a benzene dithiol molecule in the gas phase is shown in Figure 2a [19]. The right panel presents the isosurfaces of the HOMO and the LUMO wavefunctions, while the left panel shows the energy spectrum. The energy difference between the HOMO and LUMO is called the HOMO–LUMO gap, which is not only a key descriptor of the molecular electronic structure but also a critical parameter influencing molecular charge transport properties [67].

**FIGURE 2:**
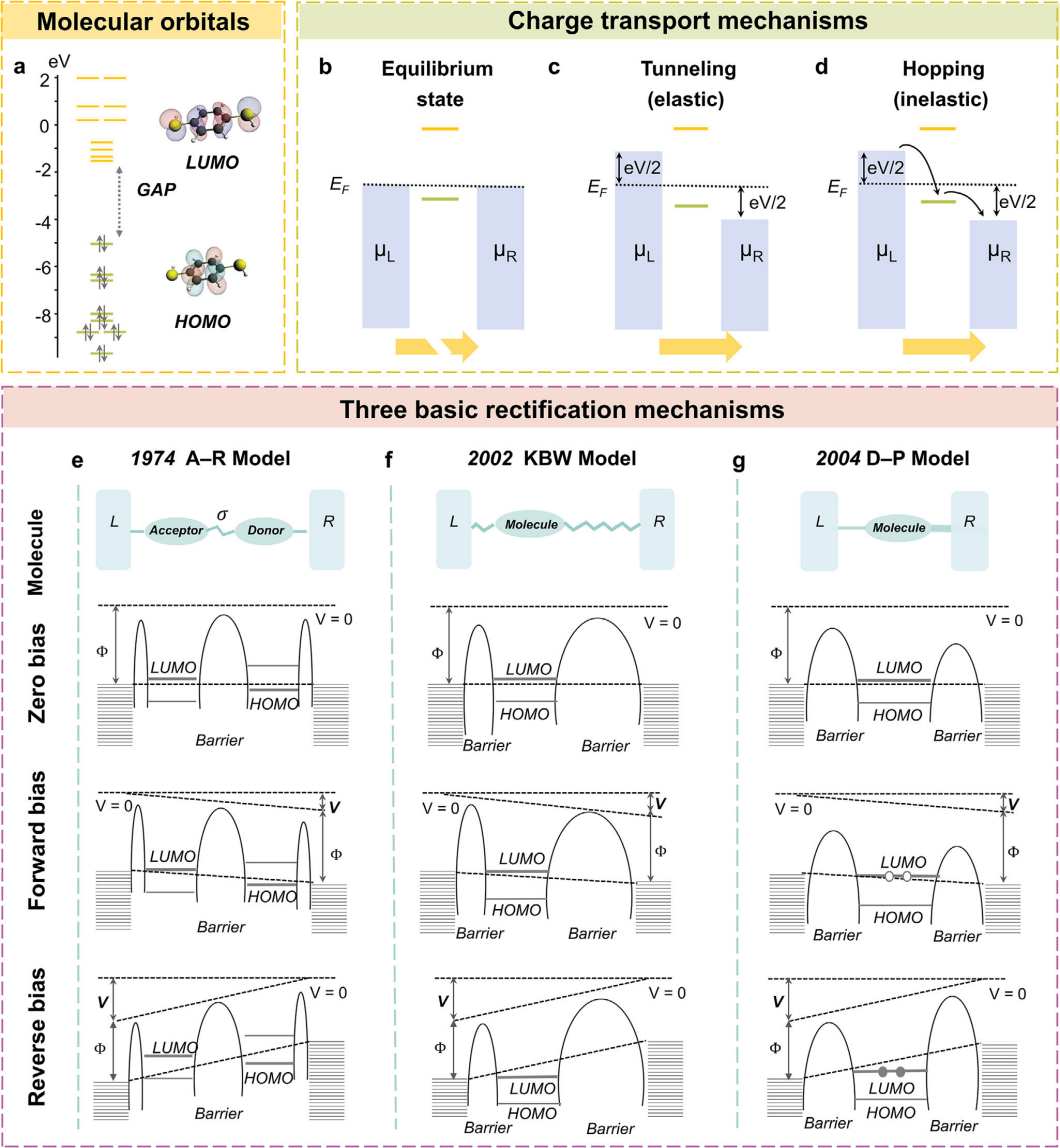
Theoretical frameworks and charge transport mechanisms. (a) Molecular energy spectrum and electronic states of the highest occupied molecular orbital (HOMO) and lowest unoccupied molecular orbital (LUMO) for a benzene dithiol molecule calculated by density functional theory (DFT). Green and yellow lines denote occupied and unoccupied orbitals, respectively. Reproduced with permission from Ref. [19]. Copyright 2015, Royal Society of Chemistry. (b–d) Energy‐level diagrams illustrating (b) the equilibrium state, (c) the coherent quantum tunneling regime, and (d) the incoherent hopping‐like charge transport regime. (e–g) Three rectification mechanisms and bias‐dependent energy‐level evolutions: (e) Aviram–Ratner (AR) model based on a donor–σ–acceptor (D–σ–A) system with an insulating spacer, where forward bias facilitates resonant tunneling by aligning the acceptor LUMO and donor HOMO, whereas reverse bias inhibits current through an increased potential threshold; (f) the Kornilovitch–Bratkovsky–Williams (KBW) model with an asymmetric tunneling barrier, where the majority of the applied bias drops across the longer insulating segment, inducing a bias‐dependent onset for resonant tunneling; and (g) the Datta–Paulsson (D–P) model, highlighting rectification driven by asymmetric molecule–electrode coupling strengths, which results in imbalanced orbital occupancy and asymmetric charging dynamics under bias. (e–g) Adapted with permission from Ref. [82]. Copyright 2012, American Chemical Society.

### 2.2 Tunneling Transport Mechanisms

When a molecule is linked to two electrodes via chemical bonding or physical adsorption, charge carriers traverse the junction through discrete MOs, while the electrodes act as reservoirs for electrons or holes [68]. Under equilibrium states, the chemical potentials of both electrodes are identical, resulting in zero net current, as illustrated in Figure 2b. Upon applying a symmetric bias voltage, the chemical potentials of the left and right electrodes shift in opposite directions, forming a bias window. If a MO lies within this window, carriers can be injected and extracted without an energy barrier, leading to substantial current flow. Conversely, if the orbital lies outside the window, transport occurs via quantum tunneling across an energy barrier, with efficiency governed by the alignment between molecular levels and electrode chemical potentials [69].

Coupling between the MOs and the electrodes induces hybridization of electronic states [70]. This coupling strength not only influences the position and broadening of the molecular energy levels, but also determines whether electrons can preserve their quantum phase during transport. Based on phase preservation, transport can be either coherent or incoherent. Coherent transport is generally dominated by elastic processes, in which electrons retain phase information without net energy exchange during transport. In contrast, incoherent transport is typically accompanied by interactions with molecular vibrations or the surrounding environment and may involve inelastic energy exchange [71]. In the coherent regime, the electron wavefunction retains phase coherence throughout the transport process. This typically occurs in short molecules with strong molecule–electrode coupling and can be described by the Landauer–Büttiker formalism, shown in Figure 2c [72, 73]. In the incoherent regime, electrons dwell on the molecule for longer times and lose phase information due to molecular vibrations or environmental interactions, which can be described by theories such as the Marcus theory [74, 75]. Such incoherent transport can proceed through sequential tunneling or hopping transport. In sequential tunneling, which is commonly observed in systems with relatively weak molecule–electrode coupling, an electron first enters the molecule from one electrode and forms a real intermediate charged state before being transferred to the other electrode. Hopping transport, by contrast, is more commonly observed in longer molecules or systems with pronounced localized states, where charge carriers migrate stepwise between different localized states or sites and often exhibit thermally activated and temperature‐dependent behavior (Figure 2d).

Another independent classification dimension is based on the relative position of the molecular levels with respect to the bias window [63]. When the molecular level aligns with the electrode chemical potentials, resonant tunneling takes place, eliminating the energy barrier and enhancing conductance. When the level lies outside the bias window, off‐resonant tunneling occurs via virtual states, and the current decreases with increasing energy mismatch. These two classification schemes are orthogonal. Coherent transport may occur under resonant conditions, often giving rise to elastic resonant tunneling, or under off‐resonant conditions as elastic off‐resonant tunneling. Likewise, incoherent transport under resonant conditions may proceed via sequential tunneling, whereas under off‐resonant conditions it may occur through thermally activated off‐resonant transport.

### 2.3 Landauer–Büttiker Formalism

In this review, we primarily adopt the Landauer framework to describe coherent tunneling in molecular junctions. Within this picture, the junction is treated as a phase‐coherent conductor, and all inelastic interactions are neglected. The current is then expressed as follows [72, 73]:

(1) $$I=\frac{2e}{h}\int_{-\infty }^{\infty }\left(T\left(E\right)\left(f_{L}\left(E\right)-f_{R}\left(E\right)\right)\right)dE$$

where *e* is the electron charge, *h* is Planck's constant, and *f*_*L*,*R*_(*E*) are the Fermi–Dirac distribution functions of the left and right electrodes. *T*(*E*) is the transmission probability for electrons of energy *E*. Within the NEGF formalism, the transmission can be expressed as [76]:

(2) $$T\left(E,V\right)=tr\left(\Gamma_{L}G^{R}\Gamma_{R}G^{A}\right)$$

where *G^R^* and *G^A^* are the retarded and advanced Green's functions of the molecular region, given by $G^{R}\;\left(E\right)=\frac{1}{E-H-\sum_{L}\left(E\right)-\sum_{R}\left(E\right)+i\delta }$ and $G^{A}\;\left(E\right)=\frac{1}{E-H-\sum_{L}\left(E\right)-\sum_{R}\left(E\right)-i\delta }$. Here, *H* is the Hamiltonian of the central region, while $\sum_{L}\left(E\right)$ and $\sum_{R}\left(E\right)$ are the self‐energies associated with the left and right electrodes. The coupling matrices $\Gamma_{L/R}\;\left(E\right)=i\left[\sum_{L/R}\left(E\right)-\sum_{L/R}^{†}\left(E\right)\right]$ quantify the orbital overlap between the molecule and the electrodes.

For a single‐level model considering only a spin‐degenerate orbital of energy *E*_0_, the transmission function reduces to a Lorentzian form [77, 78]:

(3) $$T\;\left(E\right)=\frac{\Gamma_{L}\Gamma_{R}}{{\left[E-E_{0}\right]}^{2}+{\left[\frac{\Gamma_{L}+\Gamma_{R}}{2}\right]}^{2}}\leq 1$$

This expression reveals two key principles. First, within the idealized single‐level model considered here, transmission reaches unity under resonant conditions (*E*  =  *E*_0_) when the couplings are symmetric (Γ_*L*_ = Γ_*R*_). Second, transmission increases with coupling strength, but excessive coupling leads to pronounced level broadening, diminishing the localization of molecular states and potentially degrading device functionality [79].

The Landauer–Büttiker formalism therefore provides not only a direct mathematical link between macroscopic current and microscopic transport processes, but also a unified theoretical framework for understanding how molecular energy levels, interfacial coupling, applied bias, and temperature collectively influence the performance of molecular electronic devices.

### 2.4 Rectification Mechanisms

Molecular rectification in single‐molecule devices is influenced by multiple mechanisms that arise from structural asymmetry, potential landscape variations, and interfacial coupling differences. Three prototypical theoretical models, the Aviram–Ratner (AR) model, the Kornilovitch–Bratkovsky–Williams (KBW) model, and the Datta–Paulsson (D–P) model, provide theoretical frameworks for explaining the rectification behavior of single‐molecule diodes, as schematically illustrated in Figure 2e–g [12, 80, 81, 82].

The AR model, first proposed by Aviram and Ratner, embodies the conceptual foundation of molecular diodes through a D–σ–A architecture (Figure 2e) [12]. Here, an insulating σ‐bridge spatially separates donor and acceptor units, preserving their individual electronic identities and minimizing direct coupling. Charge transport proceeds via sequential tunneling: electrons transfer from the left electrode to the acceptor, then through the donor, before reaching the right electrode. At equilibrium, the donor's HOMO lies just below the electrode Fermi level, while the acceptor's LUMO is positioned slightly above it. Under forward bias, this energy gap narrows, facilitating tunneling and reducing the conduction threshold. Conversely, reverse bias requires significantly higher voltages to align orbitals, resulting in pronounced current asymmetry. While elegant in theory, the synthetic complexity and limited tunability of the AR framework restrict its practical implementation. To address these limitations, derivative systems such as donor–π–acceptor (D–π–A) systems and donor–acceptor (D–A) block copolymers have been developed, retaining the core rectification mechanism while enhancing chemical accessibility and design flexibility [61, 83, 84, 85].

The KBW model offers a complementary perspective by attributing rectification to an asymmetric tunneling barrier interacting with a single MO, thereby dispensing with the need for distinct donor and acceptor moieties (Figure 2f) [80]. Since most of the applied bias drops across the longer insulating segment, the MO responds asymmetrically under forward and reverse biases, thereby leading to a bias‐dependent onset voltage for resonant tunneling. By adjusting the length of the insulating tails, the RRs can also be effectively tuned [22, 86]. Compared to the AR model, the KBW framework relaxes stringent energy‐level alignment requirements, thereby enhancing synthetic feasibility and experimental realization.

Distinct from the intramolecular asymmetry‐based mechanisms, the D–P model emphasizes the role of asymmetric molecule–electrode coupling in inducing rectification, even when the molecule itself is spatially symmetric (Figure 2g) [81]. Unequal coupling strengths to the electrodes result in imbalanced charging and discharging dynamics of MOs under bias, yielding direction‐dependent occupancy and consequently asymmetric current flow. Although engineering precise interfacial asymmetry remains experimentally challenging, this mechanism is frequently observed in molecular junction measurements and plays an important role in device performance [87].

Collectively, these three models capture essential mechanisms of molecular rectification from three distinct perspectives. Notably, the KBW model and the D–P model should not be regarded as completely independent descriptions. It is worth emphasizing that the D–P model, which accounts for molecule–electrode coupling asymmetry, may under certain conditions influence the intramolecular electrostatic potential profile, thereby establishing a possible connection to the asymmetric potential drop assumed in the KBW framework. In practical device engineering, effective rectification typically requires a synergistic integration of multiple mechanisms to balance structural tunability with transport performance. These three theoretical frameworks have not only laid the foundational groundwork for the field but also continue to serve as essential guiding principles for the design and optimization of molecular diodes.

## 3 Modulation Strategies

The theoretical framework of molecular rectifiers indicates that their rectification performance arises from the interplay of multiple factors. Building on rectification models and molecular transport theory, researchers have developed diverse modulation strategies, including molecular structural, interfacial, quantum interference, and external field engineering [8, 52, 88]. This section classifies these representative approaches to molecular rectification, offering methodological guidance for the design of high‐performance molecular rectifiers.

### 3.1 Molecular Structural Engineering

Modulating intrinsic molecular structures is an important strategy for enhancing rectification behavior [60]. As discussed above, both the AR and KBW models rely on molecular design to achieve rectification. However, molecular structural engineering not only encompasses traditional molecular backbone architectures, but also emphasizes precise control over torsional angles of bridging units, the length of the molecular backbone, the configuration of side chains, and the conjugation mode.

Introducing asymmetry through backbone engineering allows effective modulation of RRs. At the macroscopic level, this asymmetry manifests as an inhomogeneous potential distribution across the device, while at the microscopic level it corresponds to spatial variations in MOs. Typically, altering molecular type or length represents a commonly employed strategy. This approach is applicable to different molecular rectifier models. Length modulation is particularly relevant in KBW models and long alkyl‐chain systems, where the “tail” structure renders rectification sensitive to molecular length [89, 90, 91]. However, the effect of chain length varies among different systems. In alkylated silane SAMs, increasing molecular length reduces the RR, which correlates positively with molecular dipole strength (Figure 3a) [92]. In bipyridyl‐embedded alkanethiolate molecules, rectification occurs under positive bias for 1 ≤ *m* ≤ 3 carbon atoms on the bipyridyl's right side, whereas for 5 ≤ *m* ≤ 11, the rectification direction reverses (Figure 3b) [93]. This behavior originates from the evolution of a strongly localized frontier molecular orbital (FMO) under applied bias. Adjusting the position of the bipyridyl unit along the alkanethiolate backbone modifies the spatial distribution of the orbital, thereby influencing the rectification direction. This variation in chain‐length effects reflects differences in backbone architecture and dipole localization. In alkylated silane SAMs, dipoles become increasingly delocalized as the chain lengthens, whereas in bipyridyl‐embedded systems, the rigid bipyridyl group influences the center of orbital localization.

**FIGURE 3:**
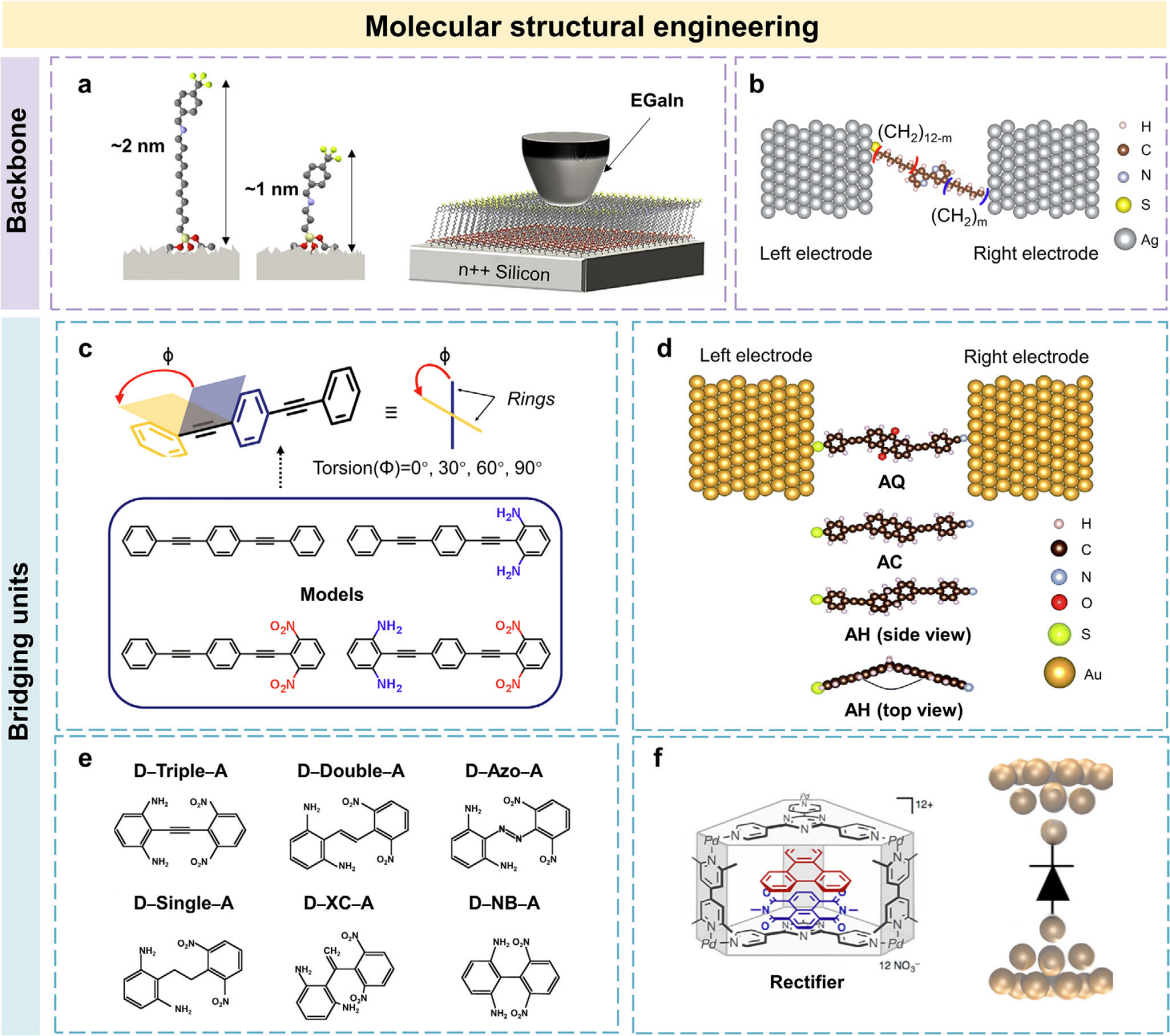
Molecular structural engineering strategies. (a) Modulation of RRs via chain length in alkylated silane self‐assembled monolayer (SAM) rectifiers. Reproduced from Ref. [92] under the CC BY 4.0 license. (b) Control of rectification direction by positioning the bipyridyl unit (*m* value) within an alkanethiolate backbone. Reproduced with permission from Ref. [93]. Copyright 2017, American Chemical Society. (c) Bridging unit engineering through torsional angle control and chemical substitution in phenylene‐based D‐B‐A systems. Reproduced with permission from Ref. [97]. Copyright 2018, American Chemical Society. (d) Device models for thiolated arylethynylene diodes utilizing cross‐conjugated (AQ), linearly conjugated (AC), and broken‐conjugated (AH) central bridges to modulate rectification. Reproduced with permission from Ref. [99]. Copyright 2017, Royal Society of Chemistry. (e) Investigation of D–B–A systems with six diverse bridge architectures (linear, cross‐conjugated, and saturated). Reproduced with permission from Ref. [100]. Copyright 2023, American Chemical Society. (f) Supramolecular rectification in a self‐assembled coordination cage containing hetero π‐stacked aromatic pairs (naphthalenediimide and triphenylene). Reproduced with permission from Ref. [101]. Copyright 2015, American Chemical Society.

In addition to altering the intrinsic molecular architecture, strategies such as elemental doping, functionalization, and side‐chain modification have also proven effective in tuning rectification (Figure 3c) [55, 94, 95, 96, 97]. Doping or functional groups modulate the MO landscape, whereas side chains can act as an internal “gate”, adjusting the alignment of MOs relative to the electrode Fermi level. Despite substantial progress in both theory and experiment, observed RRs frequently fall short of model predictions, and measured rectification direction occasionally diverges from theoretical expectations. Moreover, for large or multi‐unit molecules, synthetic complexity and molecular heterogeneity remain major challenges, underscoring the need for precise synthetic control over backbone architecture and functional group positioning to bridge the gap between molecular design and device performance.

The geometry and conjugation characteristics of bridging units also play an important role in modulating the rectification performance of molecular junctions, as illustrated by the AR model. Bridging units regulate the electronic coupling between the donor and acceptor, whereas effective electronic decoupling between these two moieties can promote rectification. Tuning the length of the molecular bridge represents an efficient strategy to weaken electronic coupling, thereby enhancing the RR, that has been confirmed in pyrene–benzene molecular junctions [98]. In addition, torsional distortions of the bridging units can partially separate the electronic states of the donor and acceptor, further promoting rectification (Figure 3c) [97]. Recent studies also compared different types of bridging structures in terms of their influence on transport behavior and rectification performance, revealing their intrinsic characteristics (Figure 3d,e) [99, 100]. As shown in Figure 3e, the investigated bridging architectures are categorized into three main types: linear conjugated bridges (triple bonds, double bonds, and azo bridges), cross‐conjugated (XC) bridges, and saturated bridges (single bonds and no bond (NB) bridges) [100]. The results indicate that cross‐conjugated bridges achieve the highest RR, reaching 2.32 under a bias of 0.15 V. This is attributed to the absence of a direct conjugation pathway between the donor and acceptor in XC systems, which confines the HOMO to the donor and the LUMO to the acceptor, thereby inducing orbital spatial separation. However, this configuration exhibits low conductance, highlighting the inherent trade‐off between RR and electrical conductivity in bridge design. Moreover, owing to the synthetic challenges associated with these structures, most studies remain at the DFT level, and the performance of such junctions falls short of that of bulk devices.

Beyond covalent connectivity, supramolecular assemblies based on π–π interactions, such as D–π–π–A systems, also exhibit notable rectification behavior (Figure 3f) [101]. Recent advances in scanning tunneling microscopy (STM) techniques have enabled precise control over the π–π stacking geometry in single‐molecule supramolecular junctions [102]. Nevertheless, the high environmental sensitivity of noncovalent interactions endows such supramolecular rectifiers with stimulus‐responsive characteristics, posing additional challenges for further enhancement of the RRs.

### 3.2 Interfacial Engineering

The Landauer formalism and the DP model both reveal, at the theoretical level, that rectification is influenced by the strength of molecule–electrode coupling. This coupling can be modulated by altering electrode materials, optimizing interfacial contact geometries, and employing tailored anchoring groups [60, 103, 104, 105]. Such approaches provide powerful means to tune interfacial coupling, thereby inducing or enhancing rectification effects.

The choice of electrode materials can influence the injection barriers and electronic coupling strength at the molecule–electrode interface [104]. Metals such as Au, Ag, Pt, Pd, and Cu have been widely used as electrodes in molecular rectifiers, and their varying work functions create distinct charge injection barriers that influence rectification [106]. Theoretical calculations by Zhang et al. showed that replacing Ag with Pt in a HOMO‐dominated molecular system can enhance the RR by orders of magnitude, as high‐work‐function metals reduce hole injection barriers and facilitate efficient carrier transport (Figure 4a) [107]. Other studies suggest that the effect primarily stems from the molecule–electrode coupling rather than energy‐level alignment, with the density of states (DOS) at the Fermi level playing a key role [108, 109, 110]. However, in either case, electrode selection can affect device rectification. Compared to metallic electrodes, carbon‐based electrodes offer unique advantages due to their excellent interfacial compatibility and tunability (Figure 4b) [111]. Coupling with molecules primarily relies on covalent bonding or π–π stacking, which are typically weaker than metallic chemical bonds. This “weak coupling” preserves the intrinsic molecular states and allows precise control over energy‐level alignment. Through doping, gate modulation, or interface functionalization, transport characteristics can be flexibly adjusted, providing greater freedom to optimize molecular rectification [55].

**FIGURE 4:**
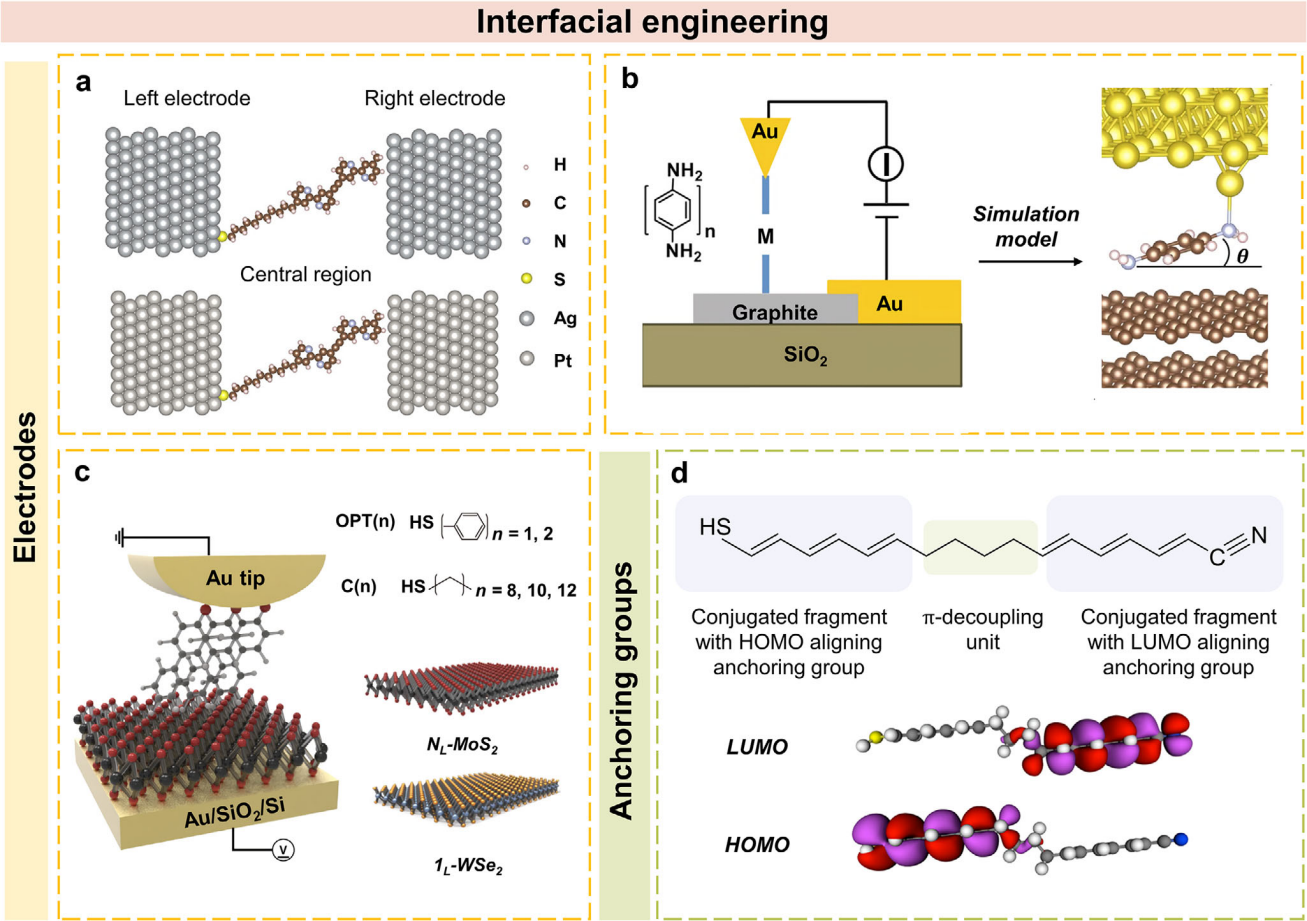
Interfacial and electrode engineering strategies. (a) Enhancement of RRs in SC_11_BIPY–C≡C–BIPY diodes by replacing Ag with Pt electrodes. Reproduced with permission from Ref. [107]. Copyright 2021, American Chemical Society. (b) Asymmetric molecular junction and its simulation model comprising a graphite substrate and an Au tip. Reproduced with permission from Ref. [111]. Copyright 2014, National Academy of Sciences. (c) Tailored diode behavior in molecular heterojunctions using two‐dimensional (2D) semiconductors (MoS_2_ or WSe_2_) as electrodes. Reproduced from Ref. [116] under the CC BY 4.0 license. (d) Rectification mechanism based on Fermi‐level pinning using asymmetric anchoring groups and π‐decoupling units. Reproduced with permission from Ref. [126]. Copyright 2015, American Chemical Society.

Beyond molecular asymmetry, electrode heterogeneity represents another effective route for enhancing RRs [112, 113, 114, 115]. Asymmetric electrode configurations can vary contact areas and interface spacing, working together with intrinsic electrode properties to shape device performance. In a landmark study, Nijhuis and colleagues achieved RRs exceeding 10^5^ in a HSC_15_Fc–C≡C–Fc (Fc = ferrocenyl) system by constructing asymmetric electrode configurations [22]. As illustrated in Figure 1, one end of the molecule formed stable chemical bonds with a metal electrode, whereas the opposite end relied on vdW interactions with an eutectic gallium–indium (EGaIn) electrode. Under forward bias, the HOMO and HOMO–1 levels entered the bias window, thereby enhancing the current. Under reverse bias, however, orbitals did not effectively enter the bias window, leading to suppressed current. In addition, the number of molecules participating in charge transport, *n*(*V*), varied with the polarity of the applied bias. When the Fc units became positively charged and experienced attractive electrostatic interactions with the negatively charged top electrode, more molecules participated in charge transport, further increasing the current. By contrast, under the opposite bias polarity, the Fc units remained neutral and interacted weakly with the top electrode, so that *n*(*V*) remained nearly unchanged. Therefore, the pronounced rectification in this system arises from the combined effects of bias‐induced energy‐level shifts and asymmetric electrode contacts. More recently, two‐dimensional (2D) semiconductors such as MoS_2_ and WSe_2_ have been incorporated as heterogeneous electrodes in organic molecule/metal interfaces, further enriching the strategies for electrode engineering (Figure 4c) [116]. The RRs can reach as high as 1.83 × 10^4^ in such systems, with the performance being highly tunable through variations in the type and number of 2D layers. Overall, molecular rectifiers leveraging electrode and interfacial asymmetry are emerging as a mainstream research direction, with device performance approaching conventional inorganic diodes [59].

Anchoring groups act as bridges between molecules and electrodes, where their conductivity and chemical nature are critical for molecular rectifiers [117, 118, 119]. Since Kushmerick et al. first demonstrated in 2004 that tuning anchoring groups can modulate rectification on demand, this approach has become a useful strategy in interface engineering [120]. Unlike molecular backbone optimization, anchoring groups act at the injection bottleneck and enable precise control of transport while preserving the intrinsic molecular electronic states.

Depending on the bonding mechanism and electronic properties, anchoring groups can be classified into two types. Covalent groups, such as –SH and Au–C, enhance interfacial electronic coupling through strong chemical bonding [121, 122]. Donor or acceptor groups, including –CN, –NH_2_, and –NO_2_, modulate energy‐level alignment via electron transfer or lone‐pair donation [123, 124, 125]. Anchoring groups with donor or acceptor characteristics are impactful in this regard. By exploiting Fermi‐level pinning, they can couple the HOMO and LUMO selectively to different electrodes. A representative example is provided by Van Dyck and Ratner, who introduced asymmetric donor and acceptor groups at opposite molecular termini and achieved RRs exceeding two orders of magnitude (Figure 4d) [126]. Under applied bias, the pinning effect drives asymmetric shifts of MOs, thereby altering tunneling probabilities in opposite bias directions and generating strong rectification behavior. Building on such design principles, recent advances in fullerene‐based molecular diodes have pushed the RRs above 10^4^, underscoring the potential of anchoring‐group design in high‐performance devices [127].

Despite these advances, some anchoring groups suffer from multiple binding configurations and weak stability, which compromise device reproducibility and lifetime [128, 129]. Coordinated efforts between theory and experiment to optimize bonding configurations, together with in situ characterization techniques, are expected to drive this strategy toward controlled, reliable, and scalable device implementation.

### 3.3 Quantum Interference Engineering

Quantum interference (QI) effects provide an alternative pathway to molecular rectification beyond conventional energy‐level alignment [45, 130]. They originate from the phase superposition of electron wavefunctions along multiple transport channels, manifesting as constructive quantum interference (CQI) that enhances current, or destructive quantum interference (DQI) that suppresses it (Figure 5a) [17, 131]. Although highly sensitive to molecular structure and symmetry, QI effects have evolved from a physical phenomenon into a rational design principle [132]. Current studies on QI effects in molecular rectifiers fall into two categories: modulation through intramolecular configuration and intermolecular interactions.

**FIGURE 5:**
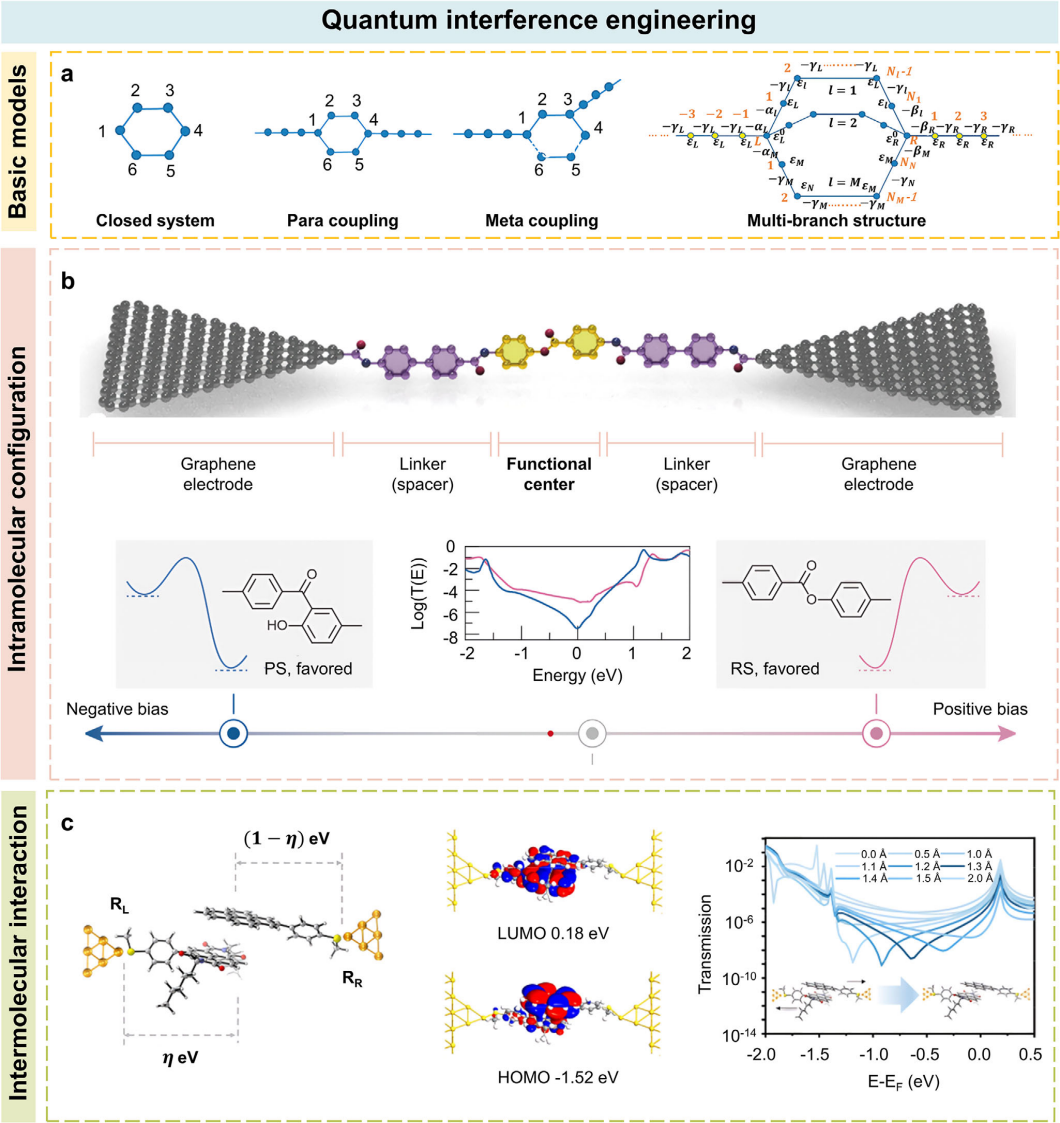
Quantum interference (QI) engineering strategies. (a) Theoretical models of QI in molecular junctions, including para‐coupling (constructive QI), meta‐coupling (destructive QI), and a generalized multibranch structure; the transmission *T*(*E*) is determined by site energies ε and hopping elements γ, where the connectivity dictates the interference patterns. Reproduced with permission from Ref. [17]. Copyright 2015, Royal Society of Chemistry. (b) Intramolecular configuration: schematic of a single‐molecule junction with a phenyl benzoate functional center. The transport symmetry is broken by the asymmetric energy profiles under opposing electric field directions, as evidenced by the shifted transmission spectra for the reactant state (RS) and product state (PS). Reproduced with permission from Ref. [46]. Copyright 2025, American Chemical Society. (c) Intermolecular interaction: supramolecular junctions with asymmetric electrostatic potentials, showing optimized frontier molecular orbital (FMO) distributions and transmission spectra at varying electrode separations. Reproduced with permission from Ref. [47]. Copyright 2025, American Chemical Society.

Intramolecular configuration control is a widely studied strategy for interference‐driven rectification, with aromatic systems serving as the primary platform. Early studies showed that asymmetric para‐meta connected diphenyl‐oligoenes intrinsically display direction‐dependent current due to bias‐sensitive coupling at the two terminals [133]. Subsequent work revealed that cross‐conjugated motifs can also introduce interference channels, enhancing RRs without compromising conductance [134]. Among these, para‐ and meta‐substituted benzenes remain the most intensively investigated [135]. Meta linkages generate DQI because of multiple destructively interfering transport pathways, strongly suppressing current, whereas para linkages exhibit CQI through coherent path superposition, thereby promoting efficient charge transmission. The central challenge, however, lies in realizing dynamic switching between para and meta configurations. A recent breakthrough came from Guo and co‐workers, who employed an electric‐field‐catalyzed Fries rearrangement to achieve reversible para‐to‐meta conversion (Figure 5b) [46]. Under forward bias, the molecule preserves the para structure and exhibits CQI. Under reverse bias, it is transformed into the meta structure, introducing DQI and switching the interference channel with bias polarity. Assisted by Lewis acid catalysis, this molecular rectifier delivered a RR exceeding 5,000 at 1.0 V, together with excellent cycling stability and reversibility. This work provides a substantive step forward in applying quantum interference to single‐molecule rectification.

Beyond molecular backbones, intermolecular interactions can also trigger and regulate QI effects [47, 132, 136]. Noncovalent forces such as π–π stacking not only stabilize supramolecular assemblies but also enable phase coupling between stacked orbitals, periodically toggling between CQI and DQI to generate rectification. A representative example was recently reported by Xu et al., who designed a D–π–π–A single‐molecule rectifier assembled from pyrene (Py) and naphthalenediimide (NDI) through π–π interactions (Figure 5c) [47]. The device exhibited pronounced bias‐dependent asymmetry in current response. More importantly, mechanical stretching induced a reversible structural reconfiguration, amplifying energy‐level asymmetry and transport‐pathway differences, thereby achieving a significant enhancement of the RR. This study provides experimental evidence that quantum interference can be modulated via intermolecular interactions, offering a new route to flexible and high‐performance molecular rectifiers.

Nevertheless, progress in this direction remains limited, constrained by the need for precise structural control at the single‐molecule and supramolecular levels, as even subtle conformational variations can alter phase coherence and interference outcomes. With advances in supramolecular chemistry and dynamic assembly strategies, the potential of this approach is beginning to unfold. More importantly, this development is transforming QI from a phenomenon into a practical design strategy for molecular rectifiers, while providing new insights into its synergistic regulation with energy‐level engineering and interfacial engineering, which enables efficient and tunable control over rectification behavior.

### 3.4 External Field Modulation

External field modulation has emerged as an important complementary strategy to dynamically tune molecular rectification. By applying physical or chemical perturbations, molecular or device systems can undergo structural reorganization, energy‐level shifts, or charge redistribution. These processes enable precise modulation of electronic rectification characteristics. Typical external fields include electric fields, temperature fields, optical fields, and chemical environment modulation [18, 25, 44, 137, 138].

#### 3.4.1 Electric Field Modulation

Electric field modulation is a well‐developed external stimulus strategy that utilizes gate electrodes to tune MO energies and interfacial chemical potentials. This process manifests as the interaction between molecules and an applied electric field, specifically the modulation of MO energies by the Stark effect [139, 140]. Building on this core mechanism, electric field modulation has evolved into two complementary paradigms. One approach is the solid‐state gate, an early strategy based on energy‐level alignment. By contrast, the more recently introduced liquid gate uses ion adsorption and desorption at the electrode surface to indirectly modulate electrode potential and tune molecular charge transport.

For instance, Perrin et al. demonstrated a gate‐tunable single‐molecule rectifier with an RR as high as 620 (Figure 6a) [141]. This study demonstrated the transition between negative differential conductance and rectification, supported by combined theoretical and experimental evidence. A negative gate bias shifts the orbital energy closer to the Fermi level. This reduces the injection barrier and thereby enhances rectification. However, the performance of solid‐state gates is dependent on device geometry and requires precise control of molecular conformation. Dual‐gate configurations can improve modulation precision, yet strong fields may distort molecular structures, limiting their practical utility.

**FIGURE 6:**
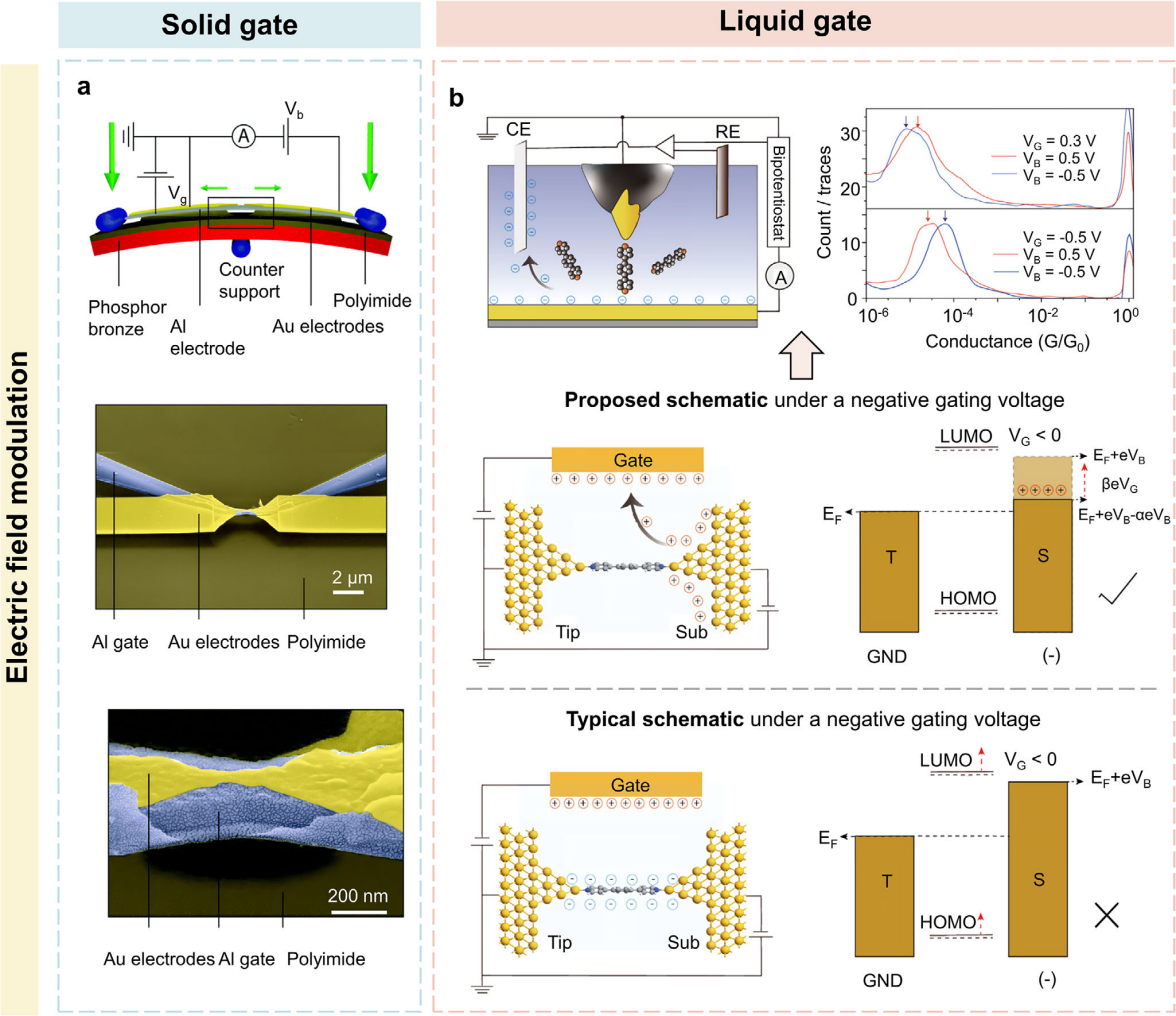
Electric field modulation strategies. (a) Solid gate modulation: schematic and scanning electron microscopy (SEM) images of a gated mechanically controllable break‐junction (MCBJ) device. Reproduced with permission from Ref. [141]. Copyright 2016, Royal Society of Chemistry. (b) Liquid gate modulation: schematic of an ionic liquid‐gated single‐molecule junction using the scanning tunneling microscope break‐junction (STM‐BJ) technique with a four‐electrode configuration. The corresponding conductance histograms and energy‐level diagrams illustrate the proposed ionic gating mechanism, where gating‐induced ion redistribution modulates the substrate Fermi level (E_F_) and the HOMO–LUMO alignment. Reproduced with permission from Ref. [27]. Copyright 2024, American Chemical Society.

Liquid gates, in contrast, provide an alternative to solid‐state gating. They provide efficient modulation at operating voltages below 1 V and are insensitive to structural variations of the device. As illustrated in Figure 6b, Wang and colleagues demonstrated in a symmetric electrode‐molecule configuration that liquid gating can even invert the rectification direction in situ [27]. Their study revealed that, within ionic liquids, gate bias modulates charge transport through the reconstruction of the interfacial electric double layer associated with the adsorption–desorption of surface‐bound ions, rather than through a simple shift of orbital energies. While the applied bias governs the initial ion adsorption, the gate voltage primarily drives ion release, resulting in precise control of rectification direction.

Together, these advances highlight the versatility of electric field modulation in enhancing rectification performance and enabling reversible and low‐power operation. However, several limitations remain. Solid‐state gating is constrained by geometric precision and molecular stability, while liquid gating, despite its adaptability, depends on complex ionic environments whose long‐term stability and integration potential are yet to be established. Addressing these challenges will be important for realizing large‐scale integration and practical applications.

#### 3.4.2 Temperature Modulation

Temperature can be used to probe the competition between tunneling and thermally activated transport mechanisms in molecular rectifiers [142]. In general, charge transport in short molecular junctions is more likely to be governed by coherent tunneling and therefore exhibits weak temperature dependence [143]. However, long molecular junctions often exhibit incoherent, thermally activated transport, which can be described within the Marcus framework [144, 145]. In classical Marcus theory, increasing temperature generally accelerates thermally activated charge transfer by promoting hopping over an activation barrier, following an Arrhenius‐type dependence on activation energy and temperature.

However, molecular length should be regarded as an empirical indicator rather than a universal criterion for determining the dominant transport mechanism. Molecule–electrode coupling, energy‐level alignment, molecular conformation, and related factors can also influence charge transport in molecular junctions. For example, long‐range tunneling has been observed in protein‐based systems spanning 7 nm, whereas thermal activation dominates transport in molecular rectifiers shorter than 3 nm [146, 147, 148, 149]. These observations do not contradict the conventional framework, but instead highlight the system‐dependent nature of temperature responses in molecular junctions. In reality, the temperature dependence of charge transport in molecular junctions exhibits complex response patterns [150]. Nevertheless, this rich dependence still provides a novel strategy for the directional optimization of molecular rectifiers.

SAM‐based devices provide a prototypical platform for temperature control, as their dimensions enable the manifestation of thermally dependent rectification. In high‐performance aromatic SAM‐based molecular rectifiers, as depicted in Figure 7a, forward bias transport exhibits thermally activated transport over a wide voltage range, whereas transport under reverse bias shows a negative temperature coefficient in certain voltage regimes [151]. This behavior is attributed to temperature‐induced entropic effects within the molecular ensemble junction. At elevated temperatures, the molecules gain greater conformational freedom, while charging of polarizable terminal groups modifies the voltage dependence of the activation energy. Consequently, the effective number of conducting molecules is modulated by both temperature and applied bias, leading to distinct temperature responses under forward and reverse bias. Beyond this specific entropic mechanism, temperature has also been shown to serve as an effective external stimulus for modulating rectification performance in other molecular junctions. For example, in benzotetrathiafulvalene (BTTF)‐based junctions, temperature modulation can enhance the RRs by up to 30‐fold (Figure 7b) [149]. In certain cases, thermal fields even enable complete reversal of rectification direction (Figure 7c) [44]. Collectively, these findings demonstrate that temperature can effectively modulate molecular rectification, offering a useful strategy for optimizing rectification performance and designing thermally responsive molecular platforms.

**FIGURE 7:**
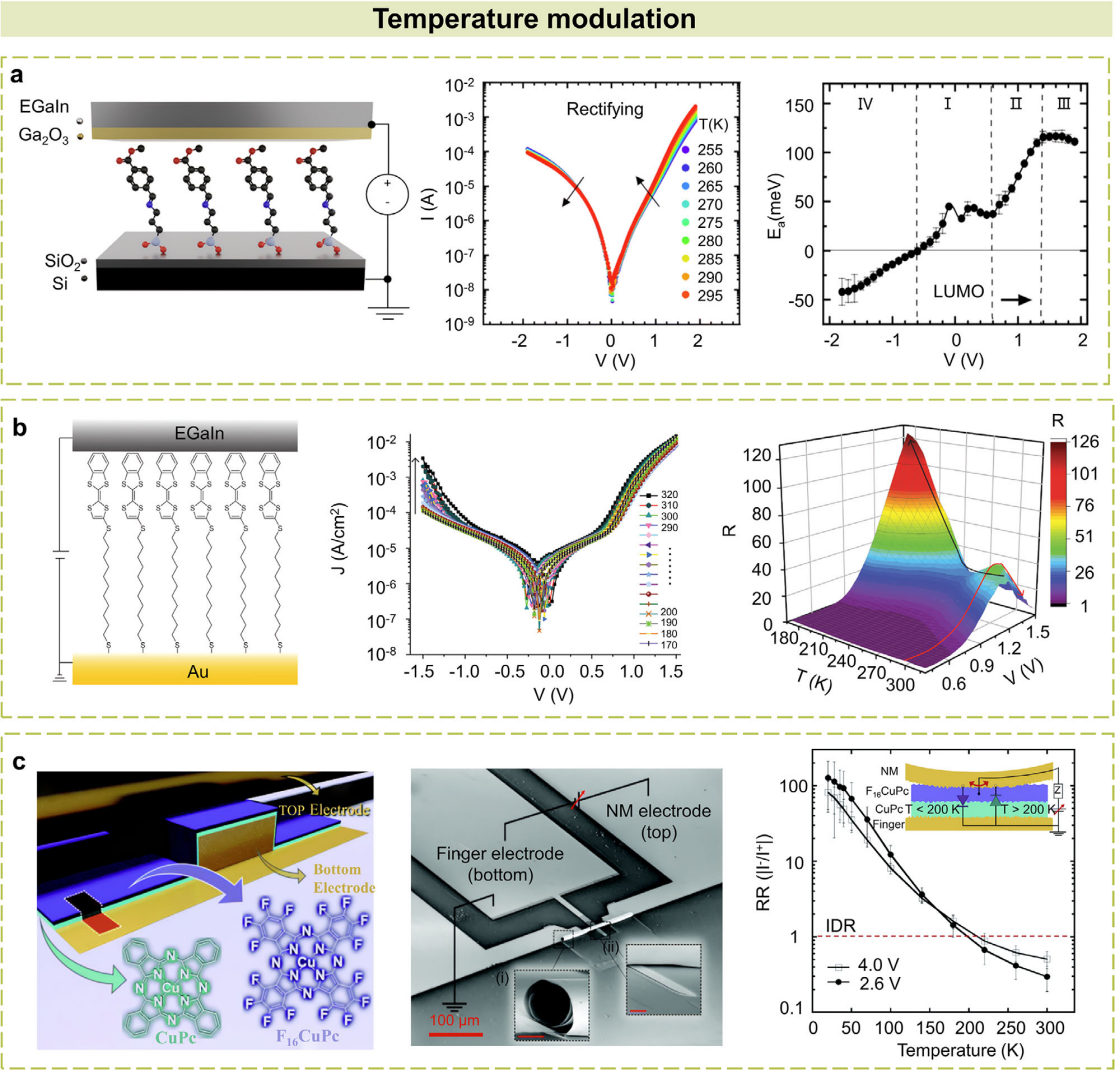
Temperature modulation strategies. (a) Device structure and current–voltage (I–V) characteristics of an (E)‐1‐(4‐carbomethoxyphenyl)‐N‐[3‐(triethoxysilyl)propyl]methanimine (CMPTM)‐based rectifying SAM, with Arrhenius plots illustrating temperature‐dependent transport across four regimes: (I) off‐resonant, (II) near‐resonant, (III) resonant, and (IV) off‐resonant. Reproduced with permission from Ref. [151]. Copyright 2024, Royal Society of Chemistry. (b) The rectifier structure, temperature‐dependent current density–voltage (J–V) curves, and three‐dimensional (3D) surface plot of the RR evolution for Au‐S(CH_2_)_11_S‐BTTF//GaO_x_/EGaIn (BTTF = benzotetrathiafulvalene; EGaIn = eutectic gallium–indium) junctions. Reproduced from Ref. [149] under the CC BY 4.0 license. (c) Molecular diode based on copper phthalocyanine (CuPc) and copper hexadecafluorophthalocyanine (F_16_CuPc) heterostructures, featuring SEM images and the average RRs with statistical deviations as a function of temperature. The equivalent circuit illustrates the temperature‐dependent device impedance (*Z*) using a thermal‐switch model. Reproduced with permission from Ref. [44]. Copyright 2020, Royal Society of Chemistry.

It is also necessary to acknowledge the limitations of thermal modulation. Elevated temperatures can induce interface reconstruction or molecular conformational changes, leading to irreversible degradation of device performance and posing challenges to operational stability. Future research could explore molecular designs incorporating reversible conformational switches or thermally responsive functional units. Such strategies may help address the limitations of single‐field modulation and facilitate the application of thermal modulation in molecular rectifiers.

#### 3.4.3 Optical Field Modulation

Light offers an attractive pathway for regulating molecular rectifiers [152]. Compared with conventional external stimuli such as electric fields or temperature, optical control provides rapid response, selectivity and reversibility [153]. In typical designs, photoresponsive molecules such as azobenzene, diarylethene and spiropyran are incorporated as the active units, enabling light‐controlled transport under irradiation with specific wavelengths (Figure 8a) [154]. The underlying mechanisms include photoisomerization, photoinduced charge transfer, and photoinduced charge accumulation. Among these, photoisomerization is a widely adopted strategy [155].

**FIGURE 8:**
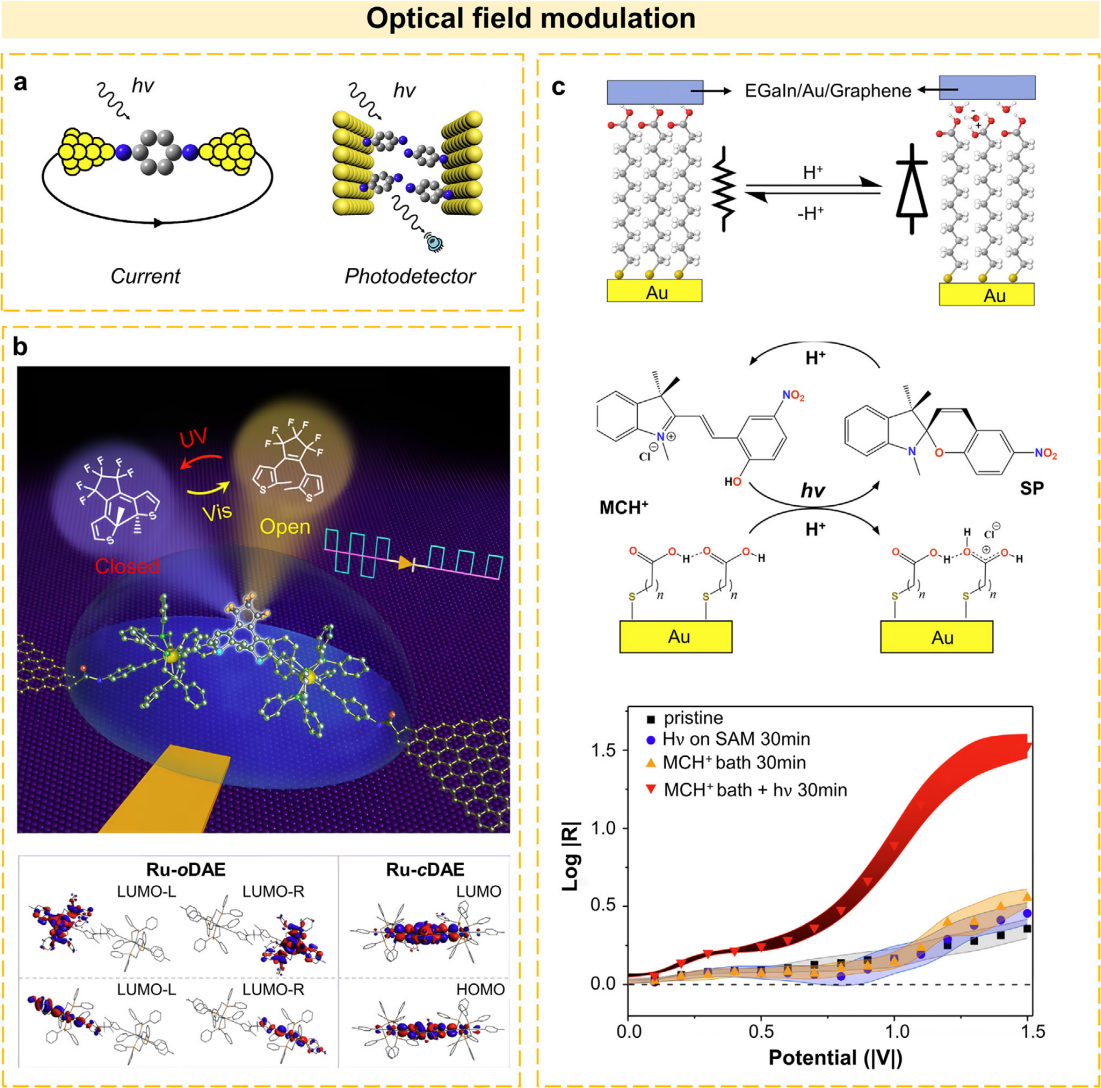
Optical field modulation strategies. (a) Graphical illustration of photo switching molecular junctions simultaneously probed by tunneling current (left) and optical signals (right). Reproduced with permission from Ref. [154]. Copyright 2024, Elsevier Ltd. (b) Graphene–Ru–DAE–graphene single‐molecule transistor with an ionic liquid gate. The schematic highlights the light‐stimulated diarylethene (DAE) isomerization between closed‐ring (Ru‐*c*DAE) and open‐ring (Ru‐*o*DAE) forms, accompanied by distinct shifts in their FMO diagrams. Reproduced with permission from Ref. [26]. Copyright 2021, American Chemical Society. (c) Photo‐controlled rectification in Au^TS^/S(CH_2_)_11_CO_2_H//EGaIn junctions. Light triggers the conversion of protonated merocyanine (MCH^+^Cl^−^) to spiropyran (SP), releasing HCl that protonates the CO_2_H‐terminated SAM. The plots of log |*R*| versus |*V*| demonstrate the resulting transition from non‐rectifying to rectifying states. Reproduced with permission from Ref. [158]. Copyright 2018, American Chemical Society.

Photochromic molecules often possess two or more stable conformations that can undergo reversible interconversion under ultraviolet (UV) or visible light [156]. Such structural switching alters molecular energy‐level alignment and molecule–electrode coupling, leading to changes in conductance and RRs. A representative example is the symmetric single‐molecule Ru–diarylethene (Ru–DAE) junction reported by Xin and colleagues (Figure 8b) [26]. Under UV or visible‐light irradiation, Ru–DAE switches between closed‐ and open‐ring configurations, and in the presence of a gate field its RRs reached more than two orders of magnitude. This approach circumvents the conventional reliance on chemical asymmetry, offering an alternative route for device design. Inspired by this concept, Wu and co‐workers used first‐principles calculations to embed photoresponsive units into molecular rectifiers, revealing optically switchable rectification and elucidating the orbital‐level mechanisms [157]. In parallel, Ai and colleagues employed spiropyran as a photoacid to reversibly switch molecular junctions between rectifying and non‐rectifying states (Figure 8c) [158]. Collectively, these studies extend the scope of optical regulation in molecular electronics and introduce functionalities such as reconfigurable circuits, which are difficult to achieve in conventional semiconductor platforms.

A further unique advantage of optical control lies in its inherent visibility. Photoinduced structural changes often generate characteristic absorption bands in the visible spectrum, accompanied by perceptible color variations that facilitate experimental readout [159, 160]. Yet significant challenges remain. Intense irradiation may cause irreversible molecular degradation, whereas insufficient light intensity may fail to drive isomerization. Balancing stability with efficiency and developing photoactive scaffolds with enhanced fatigue resistance and higher switching efficacy may support the further development of optically controlled molecular rectifiers.

#### 3.4.4 Chemical Environment Modulation

Chemical environments can regulate or enhance molecular rectification by altering the surrounding medium, such as polar solvents, ionic solutions, or humidity [161]. The key mechanism lies in the reshaping of the local electrostatic landscape and energy‐level alignment, which induces asymmetry in charge transport. Unlike electrochemical gating, which relies on external voltages and complex multi‐electrode architectures, this strategy exploits dynamic environmental variations, offering a straightforward route to control rectification. Such features are attractive for miniaturized and biocompatible molecular devices [162].

Polar media induce rectification through reconstruction of the electrode–molecule interface, forming asymmetric double layers or selective ion accumulation. A representative study by Capozzi et al. exploited the large disparity in exposed surface area between probe (∼1 µm^2^) and substrate (>1 cm^2^) electrodes to generate an asymmetric double layer in propylene carbonate (PC) solution (Figure 9a) [163]. Under negative bias, the LUMO approached the probe Fermi level, more orbitals entered the bias window, and current increased. Under positive bias, the LUMO shifted away, limiting orbital participation and suppressing current. This configuration delivered RRs above 200 at 370 mV. Control experiments showed that rectification emerged in polar solvents (PC, ionic liquids, water) and vanished in nonpolar ones such as 1,2,4‐trichlorobenzene (TCB). Even symmetric molecules like 4,4′‐bipyridine exhibited strong rectification in polar environments. These results highlight the effectiveness of ion‐induced interfacial reconstruction, further supported by theoretical analysis [164].

**FIGURE 9:**
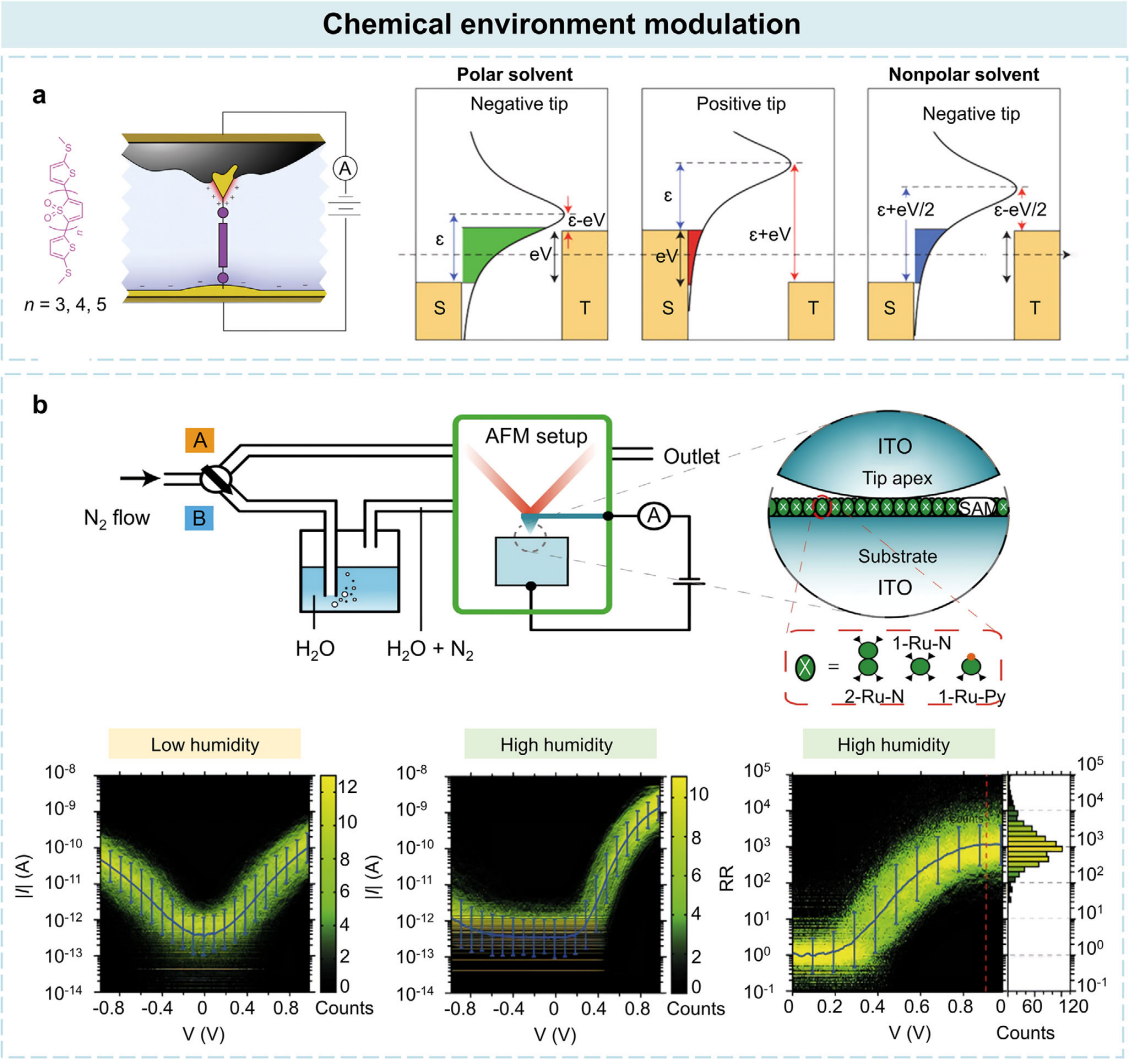
Chemical environment modulation strategies. (a) Rectification mechanism in TDOn molecular junctions. The schematic and energy‐level diagrams illustrate the influence of solvent polarity on orbital alignment. In polar media, the molecular resonance remains pinned to the substrate chemical potential, leading to asymmetric bias window overlap and high rectification. In nonpolar solvents, the resonance position is bias‐independent, resulting in symmetric transport. Reproduced with permission from Ref. [163]. Copyright 2015, Springer Nature. (b) Humidity‐modulated charge transport in ITO–molecule–ITO junctions. The conductive‐probe atomic force microscopy (CP‐AFM) setup allows for precise control of the N_2_ environment. The 2D histograms of logarithmically binned *I–V* characteristics and RRs for 2‐Ru‐N molecular junctions demonstrate the transition from low‐conductance states at 5% humidity to high‐performance rectifying states at 60% humidity. Reproduced with permission from Ref. [165]. Copyright 2018, Springer Nature.

Humidity offers another effective variable. In monolayers of dinuclear ruthenium complexes, raising relative humidity from 5% to 60% enabled reversible switching between symmetric and asymmetric current–voltage (*I–V*) responses (Figure 9b) [165]. The underlying mechanism is humidity‐dependent orbital misalignment. In dry conditions, two serially coupled orbitals are nearly degenerate, whereas high humidity induces displacement of PF_6_^−^ counterions, breaking the degeneracy and triggering orbital mismatch, thereby reshaping the tunneling barrier. These results suggest that humidity control and ionic solution regulation may share a similar physical origin associated with environment‐mediated interfacial charge reconstruction.

At a more refined level, Debye screening in electrolyte solutions provides a quantitative handle for rectification tuning. As early as 2006, Kornyshev and co‐workers theoretically predicted that RRs increase with molecular length and electrolyte concentration [166]. In dilute electrolytes, where the Debye length far exceeds the molecular length, rectification vanishes and *I–V* responses become symmetric. Recent experiments on iodo‐terminated hexathiophene (IT‐6) and circumanthracene‐based dibenzonitrile (CABD) molecules confirmed this prediction [25]. In aqueous NaF solutions, increasing ionic concentration shortened the Debye length and enhanced rectification.

Overall, chemical environment regulation provides a route to modulate molecular rectification. However, experimental outcomes are sensitive to solvent properties and humidity, while existing theoretical frameworks remain limited in their ability to rationalize the diverse responses observed across molecular systems. Future efforts should focus on establishing quantitative relationships between interfacial structure and environmental factors to support the transition from empirical adjustment to predictive design.

## 4 Fabrication Techniques

The fabrication of stable molecular junctions remains a challenge in the field of molecular rectifiers [167]. Critical hurdles include the reliable creation of nanogap electrodes, the precise incorporation of molecules into these gaps, and the realization of rectification devices with both high rectification performance and good reproducibility. Current strategies for constructing molecular rectifiers can be broadly classified into two categories, distinguished by whether the electrodes remain fixed or undergo repeated motion. The first involves dynamic junction techniques, where controlled opening and closing of electrodes allows the repeated formation of molecular contacts [48, 50]. The second category relies on static junction techniques, in which fixed electrodes are used to define molecular junctions [168].

### 4.1 Dynamic Break‐Junction Methods

Dynamic break‐junction techniques enable the reversible formation and rupture of molecular junctions by continuously tuning the electrode spacing. This approach allows the construction of single‐molecule rectifiers, while repeated opening and closing of the metallic electrodes permits thousands of junction formation events. Among these methods, MCBJ and STM‐BJ have become widely used platforms for fabricating dynamic single‐molecule junctions [48, 50, 169, 170]. Other variations, such as atomic force microscopy break junctions (AFM‐BJ) and microelectromechanical system break junctions (MEMS‐BJ), have also been reported and can be found in specialized reviews [171, 172].

#### 4.1.1 Mechanically Controllable Break‐Junction

Since its introduction in 1997, the MCBJ technique has been one of the most representative and powerful tools in molecular electronics (Figure 10a) [48]. Its operating principle is based on mechanically controlling the electrode separation: a metallic nanowire is deposited on a flexible substrate, and a pushing rod bends the substrate until the nanowire elongates and eventually fractures. This process generates atomically sharp electrodes separated by a controllable sub‐nanometer gap. Molecules equipped with anchoring groups can then bridge the gap, forming stable single‐molecule junctions whose transport properties can be probed.

**FIGURE 10:**
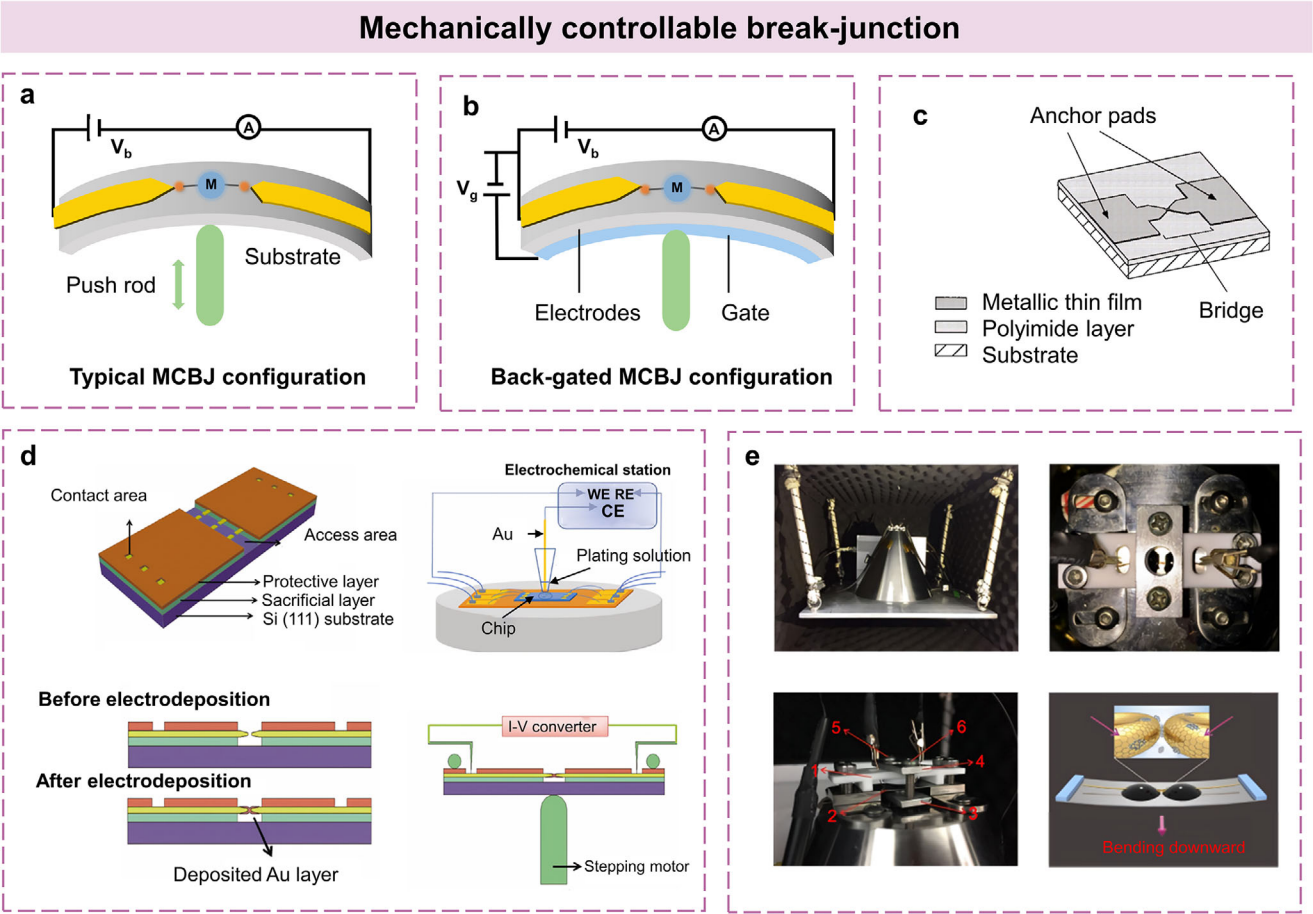
Mechanically controllable break‐junction. (a) Typical MCBJ configuration. Bending a flexible substrate yields atomically sharp electrodes with controllable gaps. (b) Back‐gated MCBJ configuration. (c) Schematic of a lithographically fabricated MCBJ chip. A polyimide layer acts as both an insulating and sacrificial layer, with a central “bridge” neck acting as a strain concentrator for electrode formation. Reproduced with permission from Ref. [176]. Copyright 1996, American Institute of Physics. (d) Electrochemically assisted MCBJ (EC‐MCBJ). The chip on a Si(111) substrate undergoes gold electrodeposition within an electrochemical station comprising working (WE), reference (RE), and counter electrodes (CE). The system, integrated with an I–V converter and a stepping motor, enables high‐throughput single‐molecule conductance measurements. Reproduced with permission from Ref. [177]. Copyright 2011, Tsinghua University Press and Springer‐Verlag Berlin Heidelberg. (e) Cross‐plane break junction (XPBJ) experimental setup. The photos and schematics illustrate the device assembly comprising a liquid cell (1), a graphene chip (2), two alloy plates (3, 4), and two screws acting as adapter pieces (5, 6). The downward bending of the graphene chip enables the investigation of single‐molecule conductance. Reproduced from Ref. [178] under the CC BY 4.0 license.

A landmark study appeared in 2005, when Elbing et al. employed the MCBJ technique to demonstrate molecular rectification at the single‐molecule level [84]. They incorporated D–A substituted molecules into gold nanogaps and observed diode‐like *I–V* characteristics with RRs approaching 10 at ±1.5 V. This work provided experimental confirmation of the AR model and highlighted the role of MO alignment in rectification. From that point on, MCBJ became widely used experimental platforms for probing single‐molecule diodes, facilitating investigation of the mechanisms underlying rectification. Beyond establishing feasibility, MCBJ has enabled systematic investigations into structure–function relationships in molecular rectifiers. Researchers have exploited its mechanical stability to investigate how molecular backbone length, substituent type, intermolecular stacking, and conformational dynamics influence rectification behavior [47, 173]. More recently, MCBJ has been coupled with external field modulation to dynamically shift MO energies [141]. To achieve gate control, various MCBJ configurations have been developed, including back‐gated, side‐gated, and electrochemically gated architectures, with Figure 10b showing a representative back‐gated configuration [174, 175]. Such experiments enabled the investigation of gate‐tunable rectification and introduced the conceptual framework of “molecular field‐effect transistors,” while providing insights into charge transport under multiple external fields.

Advances in nanofabrication have also extended MCBJ beyond traditional metallic substrates. Variants such as nanolithographically defined junctions and electrochemically assisted MCBJ chips have been reported, providing improved stability for long‐term measurements (Figure 10c,d) [176, 177]. Carbon‐based break‐junction platforms have also been developed. One example is the XPBJ method pioneered by Hong and co‐workers, as shown in Figure 10e, which uses chemical vapor deposition (CVD)‐grown graphene transferred onto metallic wires [178]. In this configuration, vdW interactions rather than conventional anchoring groups stabilize the molecular junction. RRs as high as 457 were achieved in polycyclic aromatic hydrocarbons without chemical linkers, demonstrating the potential of all‐carbon electrodes for molecular rectification [55].

Taken together, MCBJ has played an important role in the development of single‐molecule rectifiers. It has not only enabled the experimental realization of rectification at the molecular scale but has also supported investigations into the structural tuning and external‐field modulation of transport. Although limitations remain, including the inability to image molecule–electrode interfaces in real time, its stability, controllability, and ability to investigate the mechanisms underlying molecular rectification make MCBJ a valuable platform for molecular rectifier research.

#### 4.1.2 Scanning Tunneling Microscope Break‐Junction

The STM‐BJ technique originates from STM. With a piezoelectric actuator capable of controlling the probe–substrate distance at the ångström (Å) scale, STM‐BJ enables control of electrode separation and the statistical formation of single‐molecule junctions. Tao et al. first introduced this approach to single‐molecule conductance measurements, successfully probing the conductance of bipyridine (BPY) junctions through repeated stretching‐releasing cycles [50]. This development enabled statistical studies of single‐molecule charge transport and facilitated the subsequent construction of molecular rectifiers [85].

Unlike the lateral breaking process of MCBJ, STM‐BJ relies on precise vertical motion of the STM tip to form molecular junctions. During operation, the tip may contact the substrate or remain in a weakly interacting state (Figure 11a, hard‐contact and soft‐contact mode). Through rapid and repeatable stretch–release cycles, thousands of junctions can be constructed and statistically analyzed within a short time. This high‐throughput capability makes STM‐BJ suitable for statistical investigations of rectification behavior and transport mechanisms.

**FIGURE 11:**
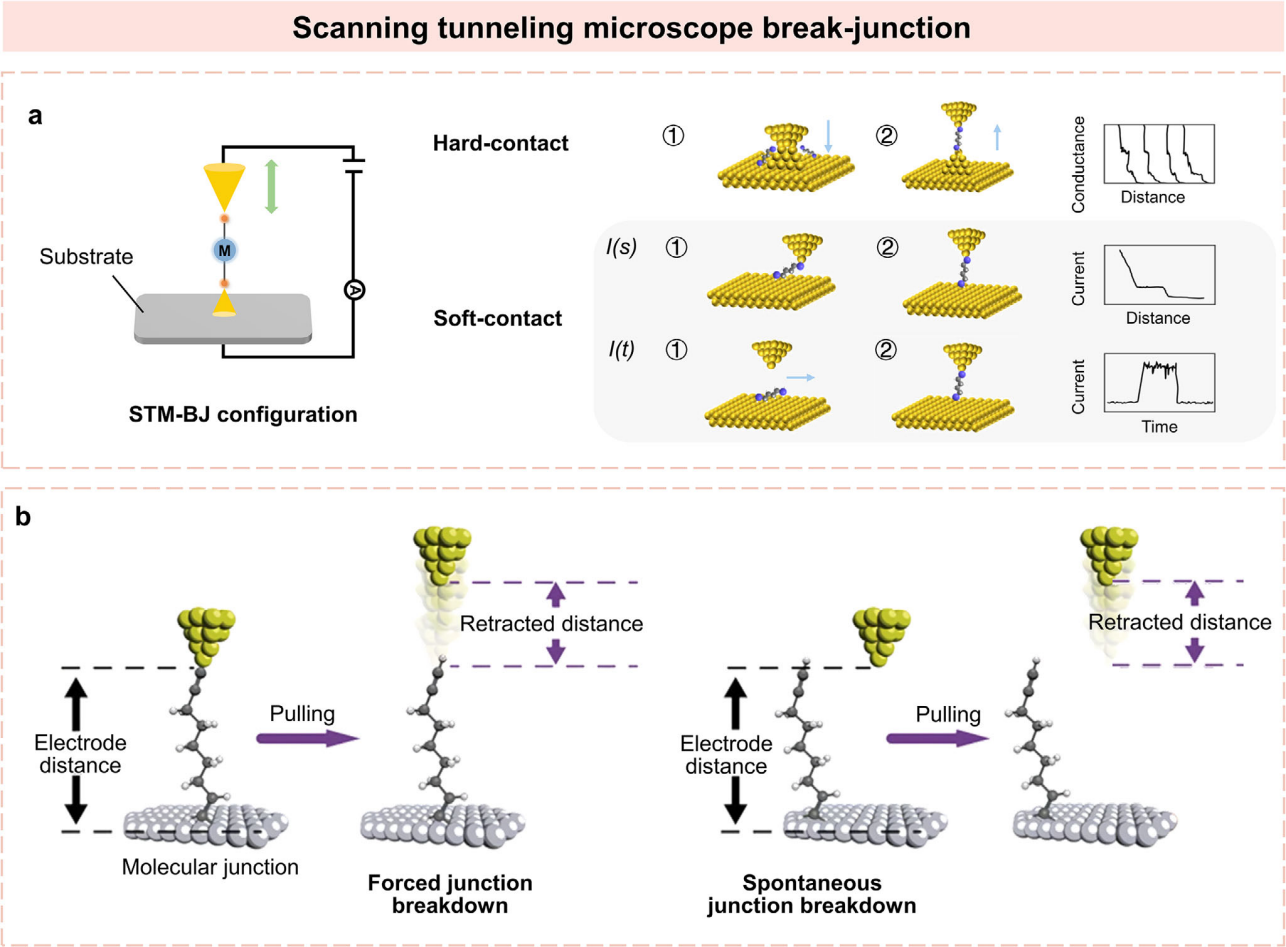
STM‐BJ techniques. (a) Schematic of the STM‐BJ setup and molecular junction evolution modes. The illustrations compare the formation and breaking processes in hard‐contact mode, *I*(*s*) mode, and *I*(*t*) mode. Reproduced from Ref. [172] under the CC BY 4.0 license. (b) STM break‐junction experiments on asymmetric gold–nonadiyne–silicon junctions. The schematics depict the pulling stage of a blinking event, contrasting the junction configurations before and after spontaneous junction breakdown. Reproduced from Ref. [182] under the CC BY 4.0 license.

In molecular rectifier studies, STM‐BJ is most commonly employed to fabricate metal–molecule–metal junctions [101, 179]. Owing to the introduction of the probe, the electrode geometry and electronic structure often exhibit inherent asymmetry, which in certain cases enhances rectification. To broaden its applicability, researchers have expanded the range of electrode materials. Beyond the conventional choices of gold (Au), silver (Ag), and platinum (Pt), electrochemical deposition has enabled the integration of other metals such as copper (Cu) and iron (Fe) onto the STM tip, thereby enriching the electrode palette [180, 181]. Meanwhile, semiconductors including silicon (Si) and gallium arsenide (GaAs) have been incorporated as substrate electrodes to construct asymmetric molecular junctions [182, 183]. This strategy diversifies electrode selection and introduces band‐structure modulation as an additional means of tuning rectification. For example, in single‐nonadiyne molecular junctions, Au top electrodes were combined with n‐doped Si bottom electrodes, where the molecule formed covalent bonds and spatial configurations on the Si surface (Figure 11b) [182]. The resulting devices exhibited improved reproducibility and mechanical stability compared with conventional Au‐based junctions. With lightly doped Si electrodes, RRs exceeded 4000, indicating the potential compatibility of single‐molecule junctions with semiconductor technologies. Similarly, carbon‐based electrodes have been integrated into STM‐BJ platforms. For instance, Kim et al. employed graphite as the bottom electrode to construct molecular junctions, providing an approach to developing heterogeneous molecular rectifiers using layered two‐dimensional materials [111].

Compared with MCBJ, STM‐BJ does not require sophisticated chip fabrication and involves a relatively simple experimental setup, making it suitable for large‐scale statistical studies with high reproducibility [105, 175, 184, 185]. However, mechanical interactions between the tip and molecules remain a challenge, limiting the mechanical stability and precise control of the molecule–electrode interface in rectifier applications. Despite these limitations, the high‐throughput capability, flexibility, and material compatibility of STM‐BJ have established it as a key experimental methodology driving mechanistic insights and performance optimization in molecular rectifiers.

### 4.2 Static Molecular Junction Techniques

Static molecular junctions are constructed by assembling target molecules within a fixed electrode gap. Compared with dynamic junctions, this strategy eliminates the need for continuous control of electrode spacing, which can provide greater structural stability and compatibility with semiconductor processing. Static molecular junctions are generally classified into three types: SAMs, carbon‐based junctions, and electromigrated break junctions (EBMJs) [26, 186, 187]. Among them, SAMs and carbon‐based junctions can offer advantages in reproducibility, interfacial control, and device scalability and will be the focus of this review. Detailed progress on EBMJs has been summarized elsewhere [188].

#### 4.2.1 Self‐Assembled Monolayers

SAM‐based junctions represent a controllable and stable strategy for ensemble molecular devices. The principle relies on anchoring groups that self‐assemble into an ordered monolayer on the bottom electrode, followed by deposition of a top electrode to complete the junction, as depicted in Figure 12a. Although this method does not provide precise positioning at the single‐molecule scale, it offers benefits in device uniformity, thermochemical stability, and large‐scale integration. These features have made SAMs a widely used platform for reproducible and scalable studies of molecular rectifiers.

**FIGURE 12:**
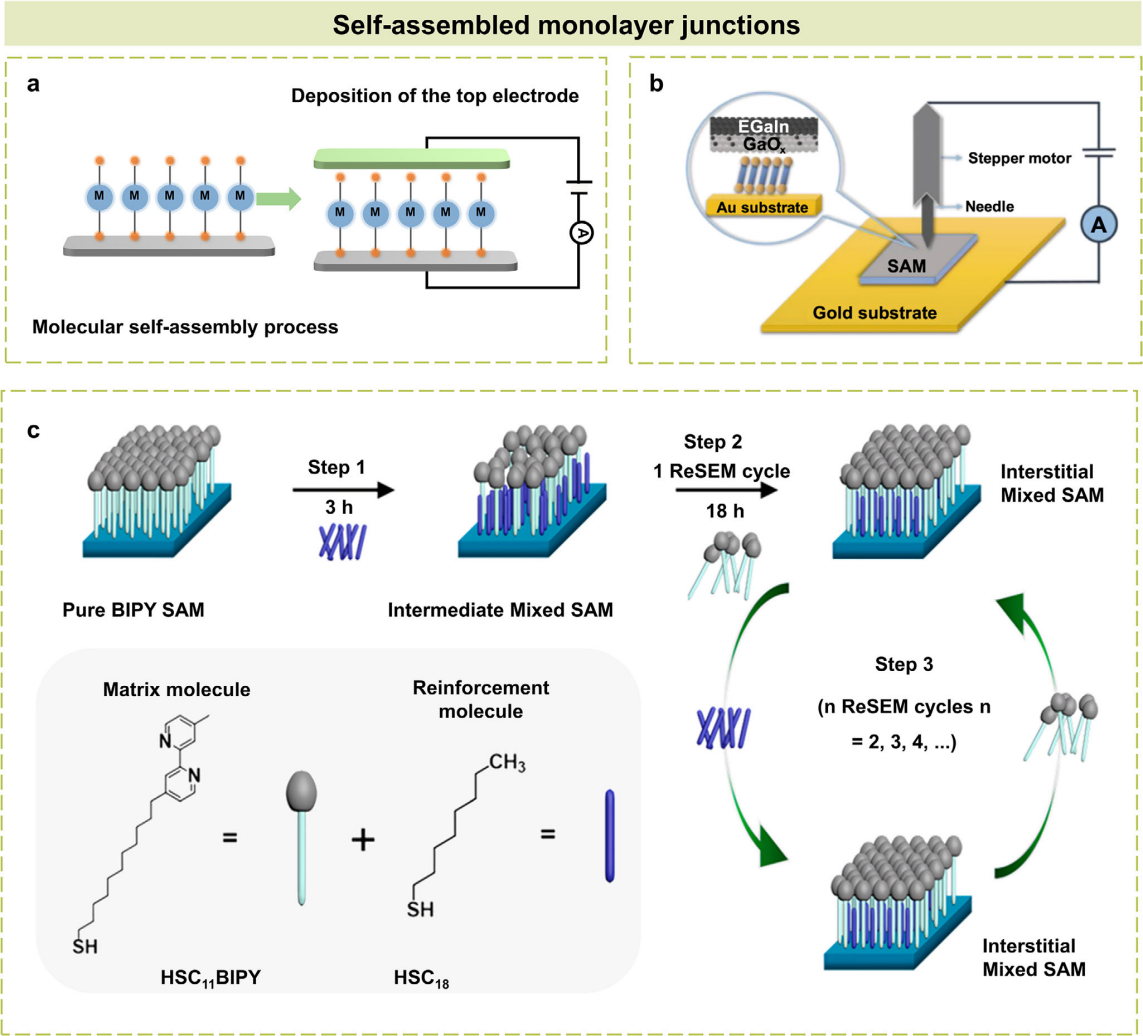
Self‐assembled monolayer junctions. (a) Typical SAM‐based molecular junction. The architecture relies on anchoring groups that self‐assemble into an ordered monolayer on a bottom electrode, followed by the soft‐contact deposition of a top electrode to complete the junction. (b) EGaIn/SAM/Au junction. The liquid metal electrode conforms to the SAM surface, providing a nondestructive contact that reduces the risk of short circuits. Reproduced with permission from Ref. [194]. Copyright 2019, Wiley‐VCH Verlag GmbH & Co. KGaA, Weinheim. (c) Formation of mixed SAMs through repeated surface exchange of molecules (ReSEM). The schematic illustrates repeated surface exchange between functional matrix molecules and skinny reinforcement molecules, together with representative molecular structures used for constructing electrically stable mixed SAMs. Adapted with permission from Ref. [201]. Copyright 2021, American Chemical Society.

Early attempts to fabricate SAM junctions relied on deposition of metals such as Au or Ti [189]. This often punctured the organic monolayer, causing short circuits and poor reproducibility [190]. To overcome this challenge, researchers explored alternative strategies, including the introduction of buffer layers such as graphene, conductive polymers, or ultrathin Al_2_O_3_, as well as soft‐contact methods such as transfer printing or nanoimprinting [191, 192]. However, these approaches can involve challenges associated with interfacial control, fabrication yield, and experimental complexity, which have limited their broader adoption. A notable development came with the introduction of EGaIn electrodes [193]. EGaIn itself has a high surface tension, but exposure to air leads to the formation of a thin GaO_x_ layer on its surface. This oxide layer stabilizes the electrode geometry and works together with the mechanical compliance of the liquid‐metal core, allowing EGaIn to form soft contacts with SAMs and reduce the risk of puncturing the molecular layer during metal deposition. A typical process involves assembling a functional SAM on a gold substrate, followed by gentle contact with liquid EGaIn as the top electrode (Figure 12b) [194]. Since its introduction, EGaIn has facilitated the development of SAM‐based junctions as an experimental platform that enables statistically reliable measurements across multiple junctions. Reported RRs typically fall in the range of 10–10^3^, and can exceed 10^5^ with careful molecular design and interfacial optimization, reaching values comparable to those reported for certain semiconductor rectifiers in terms of RR under specific conditions [112, 195, 196]. The EGaIn platform enables statistical validation and quantification of molecular rectification, improving the reliability and comparability. However, when evaluating the transport properties of EGaIn/SAM junctions, the nominal or geometrical contact area used to calculate the current density may differ from the effective electrical contact area involved in charge transport. Environmental conditions, interfacial wettability, the morphology of the native oxide layer on EGaIn, and the microscopic roughness of the SAM substrate can affect the interfacial contact state and junction reproducibility, and the effective electrical contact area may be smaller than the geometrical area [197, 198]. This difference should be considered when comparing current densities and rectification performance among different molecular systems.

Beyond the optimization of top‐electrode contacts, improving the intrinsic electrical stability of SAMs has also become an important direction. Variations in molecular orientation, packing density, and residual defects can lower the breakdown voltage and introduce device‐to‐device variability [199, 200]. To address this issue, repeated surface exchange of molecules (ReSEM) has recently been developed as a supramolecular strategy for constructing electrically stable mixed SAMs [57]. In this method, a pure SAM composed of functional matrix molecules is first formed on a metal substrate and then subjected to repeated surface exchange with skinny reinforcement molecules. These reinforcement molecules fill intermolecular interstices and nanoscale pinhole defects between the matrix molecules, forming a more densely packed interstitially mixed SAM. As a result, the resulting mixed SAM can improve the packing quality and breakdown voltage while preserving the original electronic function of the matrix molecules. For example, in a SAM‐based molecular rectifier composed of 2,2′‐bipyridyl‐terminated *n*‐undecanethiol (HSC_11_BIPY), the incorporation of *n*‐octanethiol (HSC_8_) reinforcement molecules through ReSEM increased the breakdown voltage from 1.4 to 3.3 V without degrading the rectification function (Figure 12c) [201]. The increased breakdown voltage further widens the accessible bias window, allowing deeper MOs to participate in charge transport and enabling bias‐controlled disappearance and even polarity inversion of rectification. These results indicate that ReSEM can enhance the electrical stability of SAMs and expand the accessible high‐bias functionality of SAM‐based molecular junctions.

As a further extension of ordered monolayer platforms, MOCs have recently emerged as a platform for static molecular junctions. Unlike the stochastic assembly of conventional SAMs, MOCs provide a long‐range ordered molecular lattice that can provide improved structural uniformity and stability. For instance, Li et al. demonstrated the growth of high‐quality monolayer C_6_‐DNTT single crystals over millimeter scales using solution‐shearing epitaxy [24]. By integrating these MOCs with vdW metal contacts, they fabricated molecular diodes that achieved an RR of 5 × 10^8^ and an ideality factor close to unity. This approach may help mitigate reproducibility and stability issues in molecular‐layer devices, thereby complementing SAM‐based platforms.

Nevertheless, these platforms possess intrinsic limitations as ensemble devices. Their electrical responses generally originate from the averaged contribution of a large number of molecular channels, rather than the intrinsic transport properties of individual molecules. In addition, local variations in molecular orientation, interfacial structure, and environmental conditions may still introduce statistical dispersion across large‐area junctions. As such, SAMs and their extended platforms are particularly suitable for statistical validation and performance evaluation of molecular rectification, but are less suited for resolving detailed transport mechanisms at the single‐molecule level.

#### 4.2.2 Carbon‐Based Junctions

Carbon‐based molecular junctions have emerged as an alternative that overcomes key limitations of metal electrodes. Carbon nanomaterials such as carbon nanotubes (CNTs) and graphene combine distinct electronic structures, interfacial compatibility, and mechanical flexibility [202, 203, 204]. These features can reduce interfacial defects and suppress MIGS, allowing molecular level alignment that is closer to theoretical predictions. In contrast to SAMs, carbon‐based junctions can more readily achieve static single‐molecule configurations, providing an experimental route to explore intrinsic charge transport.

Fabrication of carbon‐based junctions typically involves creating nanogaps within carbon electrodes, followed by the insertion of functional molecules. Common approaches include electrical breakdown, local oxygen plasma etching, and focused ion beam (FIB) milling, with oxygen plasma etching widely because of its relatively straightforward operation and ability to control the gap size [205, 206, 207]. Among these, the physical strategy of current‐induced breakdown allows for the creation of nanogaps that can be further refined through electron‐beam irradiation, as demonstrated in the fabrication of DNA‐based molecular devices (Figure 13a). This method facilitates in situ observation and gap adjustment to match the dimensions of the target biomolecules. Plasma treatment generates nanogaps terminated with carboxyl groups, which can covalently link to amine‐terminated molecules via amide bonds, facilitating stable molecular integration. More recently, the combination of plasma etching with lithography and electrical breakdown has enabled high‐yield fabrication of CNT and graphene nanogap electrode arrays, supporting the large‐scale integration of molecular rectifiers [208, 209].

**FIGURE 13:**
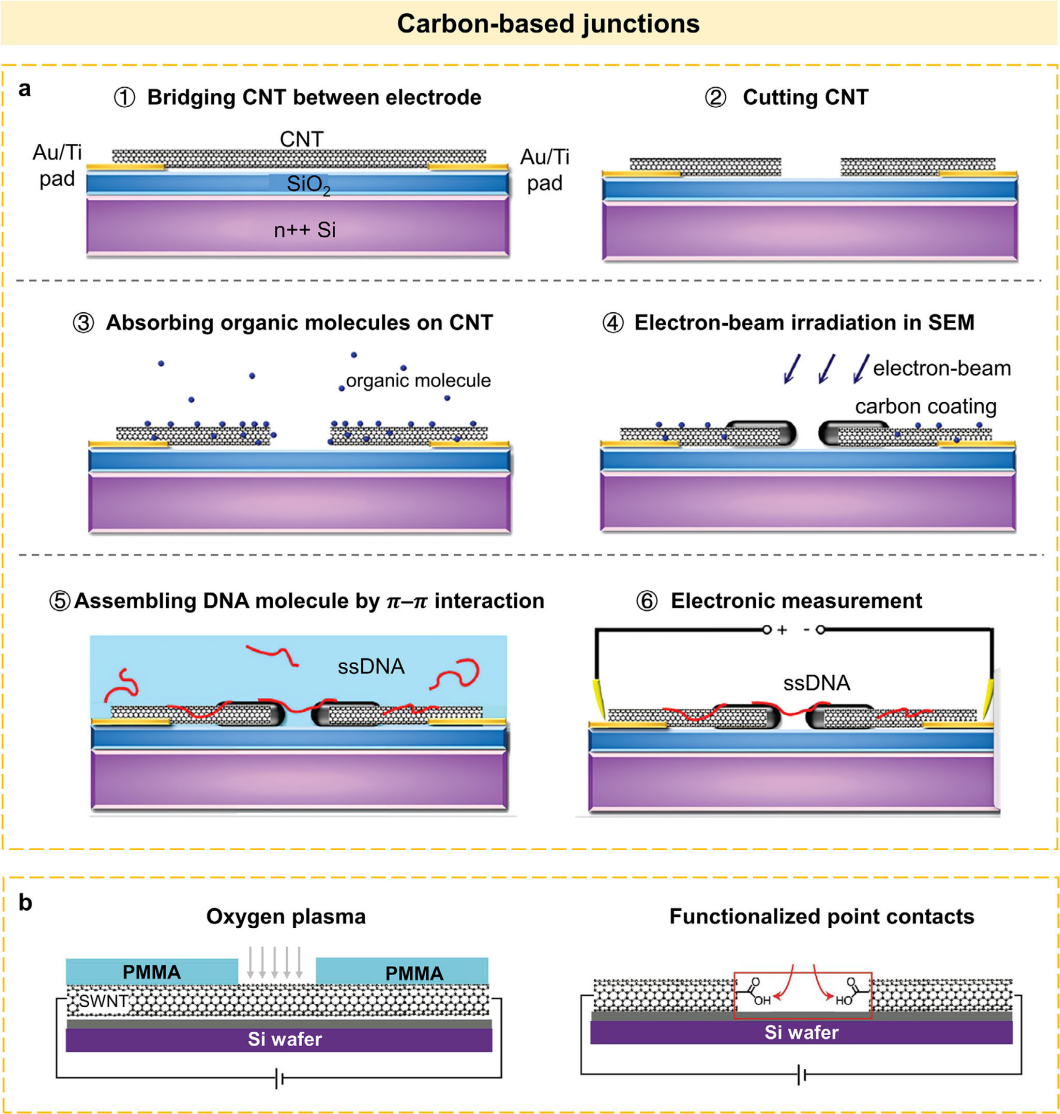
Carbon‐based junctions. (a) Fabrication of carbon nanotube (CNT) electrodes with controlled nanogaps for DNA electronic devices: the process involves bridging a CNT between Au/Ti electrodes, followed by cutting via current breakdown and electron‐beam irradiation to refine the gap, enabling the assembly and electronic measurement of DNA molecules. Reproduced with permission from Ref. [205]. Copyright 2008, American Chemical Society. (b) Controlled cutting of single‐walled carbon nanotubes (SWNTs) via oxygen plasma. A sub‐10 nm window is defined in a poly(methyl methacrylate) (PMMA) mask using electron‐beam lithography, followed by oxygen plasma etching to create carboxylate‐functionalized point contacts for molecular bridging. Reproduced with permission from Ref. [210]. Copyright 2008, American Chemical Society.

Taking CNT‐based molecular rectifiers as a representative example, a typical chemical fabrication process based on oxygen plasma etching is illustrated in Figure 13b [210]. Large‐area CNT is first synthesized by chemical vapor deposition and transferred onto a silicon substrate. A thin layer of poly(methyl methacrylate) (PMMA) is then spin‐coated and patterned using electron‐beam lithography to define micro‐ and nanoscale electrode features. Oxygen plasma etching combined with controlled electrical breakdown generates nanogap point electrodes terminated with carboxyl groups. Target molecules are subsequently assembled into the gap and covalently coupled to the CNT electrodes, yielding a robust molecular rectifier. This strategy can offer advantages in interfacial coupling and energy‐level alignment and provides an experimental approach that supports consistent junction fabrication and large‐area device integration.

Carbon‐based junctions can offer interfacial compatibility, efficient molecular integration, mechanical flexibility, and scalability [211]. However, challenges remain, particularly the complexity of material synthesis and transfer, as well as limited uniformity of large‐area electrodes. Despite these challenges, carbon electrodes expand the range of materials available for molecular rectifier fabrication and may support the scalable fabrication of molecular electronic devices.

## 5 Characterization Techniques

The performance of molecular rectifiers depends not only on the synthesis of individual molecules but also on the assembly of the complete device. Consequently, their characterization often involves characterization across multiple scales, from individual molecules to complete devices [212]. In this section, techniques for probing molecular and interfacial structures are first reviewed, followed by methods for quantifying rectification performance via electrical measurements. Finally, approaches for characterizing the electronic structure are discussed. Together, these methods are used to investigate device behavior and charge transport in molecular rectifiers.

### 5.1 Structure and Interface Characterization

The performance of molecular rectifiers depends not only on the intrinsic structure of the molecules but also by their interactions with electrode interfaces [213]. Molecular composition and conformation, electrode geometry, the size of nanogaps, and the nature of anchoring and chemical bonding at the interface influence charge transport efficiency and RRs. Consequently, structural and interfacial characterization of molecular rectifiers needs to span multiple length scales, from the molecular to the device level.

At the molecular level, structural integrity and chemical purity are prerequisites for reliable device fabrication. Commonly employed techniques include nuclear magnetic resonance (NMR) spectroscopy, high‐resolution mass spectrometry (HRMS), infrared spectroscopy (IR), and differential scanning calorimetry (DSC) [214, 215, 216, 217]. NMR provides detailed information on the molecular backbone, functional groups, and the chemical environment of substituents, whereas HRMS provides information on molecular mass and composition. IR spectroscopy and DSC focus on conformation and thermal stability. IR detects conformational changes induced by external stimuli, such as temperature or light, via characteristic vibrational peaks of functional groups. DSC evaluates the suitability of molecules for device integration by measuring melting (T_m_) and decomposition temperatures (T_d_). These methods are used to evaluate the identity, purity, conformation, and thermal stability of target molecules before interfacial assembly and electrical measurements.

At the device level, characterization primarily addresses two key aspects: the geometry and dimensional control of nanogap electrodes, and whether molecules form stable and ordered assemblies on the electrode surface. Techniques commonly employed include atomic force microscopy (AFM), scanning electron microscopy (SEM), STM, high‐angle annular dark‐field scanning transmission electron microscopy (HAADF‐STEM), energy‐dispersive X‐ray spectroscopy (EDS), X‐ray photoelectron spectroscopy (XPS), angle‐resolved XPS (ARXPS), and ellipsometry [218, 219, 220, 221, 222, 223, 224, 225]. AFM, SEM, and STM probe electrode morphology and the spatial distribution of nanogaps at the microscale. HAADF‐STEM and EDS reveal elemental composition and structural details of the molecule–electrode interface at the atomic scale. XPS and ARXPS provide insights into interfacial chemical states and the bonding modes of anchoring groups, while ellipsometry measures monolayer thickness and coverage. Together, these complementary techniques map the structural landscape of interfaces across multiple scales.

The focus of characterization is influenced by device fabrication strategies. In SAM systems, molecular order and film uniformity are important for device stability. AFM can be used to assess monolayer height distribution and local defects, while ellipsometry evaluates large‐area film thickness and coverage. XPS is used to confirm chemical bonding at the interface. When additional information on molecular stacking orientation or long‐range order is required, X‐ray diffraction (XRD) can provide valuable insight [101, 226]. In addition, near‐edge X‐ray absorption fine structure (NEXAFS) spectroscopy can probe the tilt angles and backbone orientation of molecules, offering complementary atomic‐scale information on molecular conformation at molecule–electrode interfaces [227]. Figure 14a presents the molecular structures of *n*‐mercaptoalkanoic acids and the device architecture of a molecular junction composed of a C11 SAM on an Au substrate with a Ga_2_O_3_/EGaIn top electrode [228]. Figure 14b shows AFM topography images comparing the bare Au substrate and the 11‐mercaptoalkanoic acid (C11) SAM, illustrating the formation of a uniform molecular layer. Ellipsometry data (Figure 14c,f) exhibit a linear dependence of film thickness on chain length and a slight increase in thickness after deprotonation. Reflection‐Absorption Infrared Spectroscopy (RAIRS) in the C–H and C = O regions (Figure 14d,g) confirms the crystalline‐like molecular ordering and the conversion of COOH to COO^−^ groups. Additionally, high‐resolution C 1*s* and K 2*p* XPS spectra (Figure 14e,h) reveal the chemical composition of the terminal groups and the stoichiometric coordination of alkali metal ions after deprotonation. Thus, AFM, ellipsometry, and XPS are commonly combined to characterize SAM‐based molecular junctions.

**FIGURE 14:**
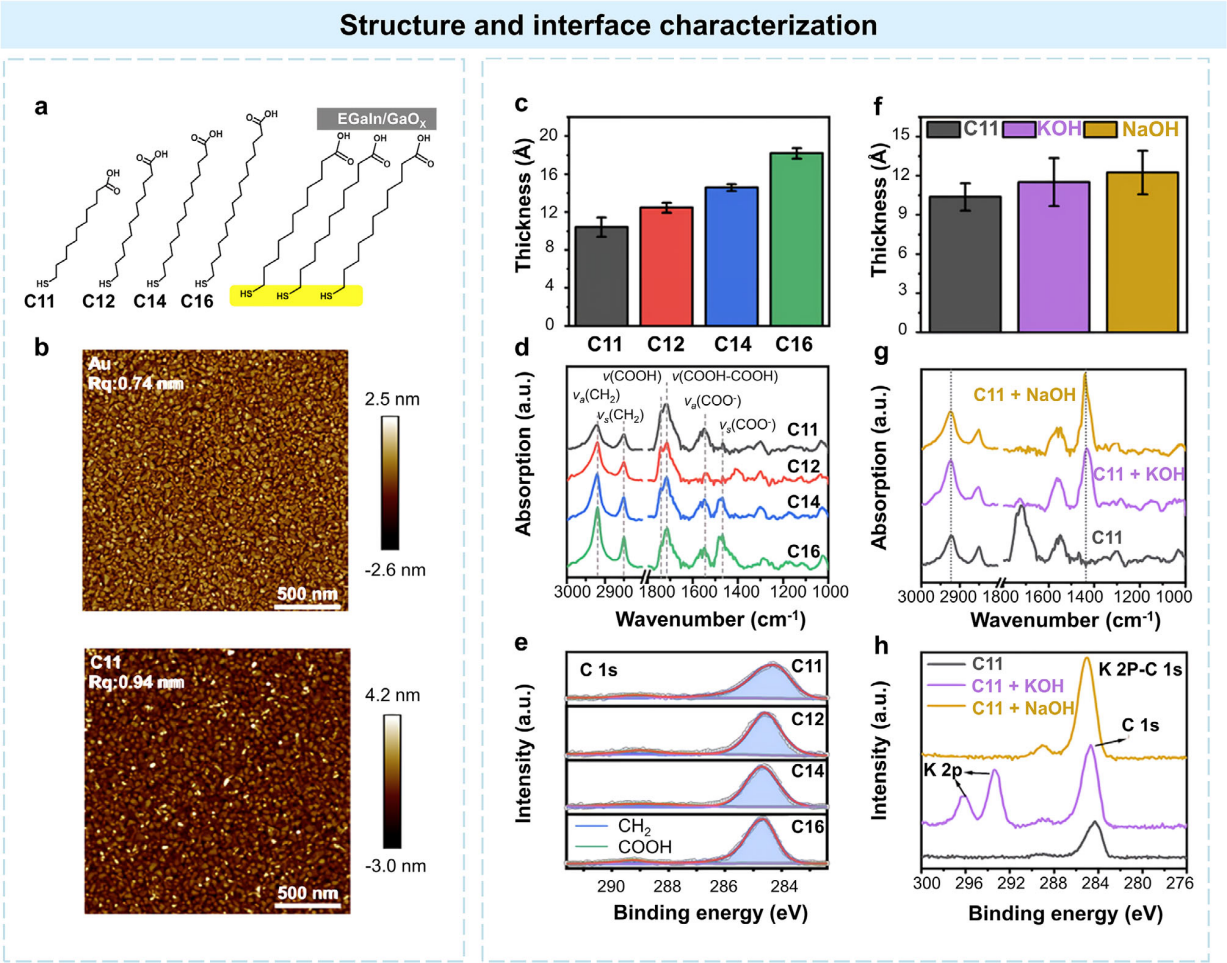
Structure and interface characterization. (a) Molecular structures and device architecture. The series includes *n*‐mercaptoalkanoic acids and a schematic illustration of the molecular junction formed by a C11 SAM on an Au substrate with a Ga_2_O_3_/EGaIn top electrode. (b) Atomic force microscopy (AFM) images compare the Au substrate and the 11‐mercaptoalkanoic acid (C11) SAM, illustrating the formation of a uniform molecular layer. (c,f) Ellipsometry data showing the linear dependence of film thickness on chain length and the slight thickness increase after deprotonation. (d,g) Reflection‐absorption infrared spectroscopy (RAIRS) in the C–H and C = O regions confirming the crystalline‐like molecular ordering and the conversion of COOH to COO^−^ groups. (e,h) High‐resolution C 1*s* and K 2*p* XPS spectra illustrating the chemical composition of the terminal groups and the stoichiometric coordination of alkali metal ions after deprotonation. Reproduced with permission from Ref. [228]. Copyright 2021, American Chemical Society.

For carbon‐based electrodes, characterization priorities differ. The structural integrity and defect density of graphene or carbon nanotubes influence molecular‐electrode coupling. Raman spectroscopy (RS) can be used to evaluate layer number, defect density, and local strain, whereas SEM and AFM provide information on surface uniformity and molecular coverage [46, 55].

In contrast, in break‐junction devices prepared via STM‐BJ or MCBJ, the interface is not statically stable but undergoes continuous formation–rupture–reformation during electrical measurements. In such systems, conventional imaging and spectroscopic characterization are of limited utility; instead, statistical analysis of thousands of conductance traces is often employed to identify frequently occurring molecular junction configurations. Here, electrical measurements provide indirect information on junction configurations, with microscopy and spectroscopy acting as auxiliary validation tools.

Overall, structural and interfacial characterization of molecular rectifiers commonly combines multiple techniques across different length scales. Looking forward, the development of in situ dynamic characterization techniques, capable of capturing real‐time molecular conformations and interfacial states under operational conditions remains important [172, 229, 230]. Such advances will provide information on the dynamic mechanisms underlying rectification and inform the design of molecular electronic devices.

### 5.2 Electrical Characterization

The functional performance of molecular rectifiers is reflected in their electrical response, making electrical characterization an important step for evaluating device rectification and application potential. Variations in molecular structure or interfacial configuration can change conductance, and molecular junctions constructed using different methods may exhibit different rectification behaviors [231]. Establishing standardized and reproducible protocols is therefore important to enable meaningful comparisons and mechanistic insights.

In dynamic break‐junction techniques, conductance histograms are typically employed to identify stable molecular configurations, followed by *I–V* measurements under those conditions [50]. A well‐defined conductance plateau supports reproducible statistical analysis of single‐molecule junctions. By repeatedly forming and breaking the junction, thousands of conductance traces can be rapidly collected, generating one‐dimensional (1D) or two‐dimensional (2D) conductance histograms (Figure 15a–c) [232, 233]. A 1D histogram displays the frequency of different conductance channels, with the horizontal axis commonly plotted as log(G/G₀) (Figure 15b); peak positions correspond to the stable molecular conformations or molecule–electrode binding motifs, providing a standard reference for typical conductance states [234]. 2D histograms, such as conductance–distance maps, offer additional insight into junction stability during elongation, revealing correlations between molecular orientation, electrode polarity, and rectification behavior (Figure 15c) [233]. Once stable conductance plateaus are observed, voltage sweeps are applied to record *I–V* characteristics (Figure 15d). By aggregating numerous traces into *I–V* histograms and fitting the data statistically, an average response can be obtained, from which RRs under positive and negative bias are calculated (Figure 15e).

**FIGURE 15:**
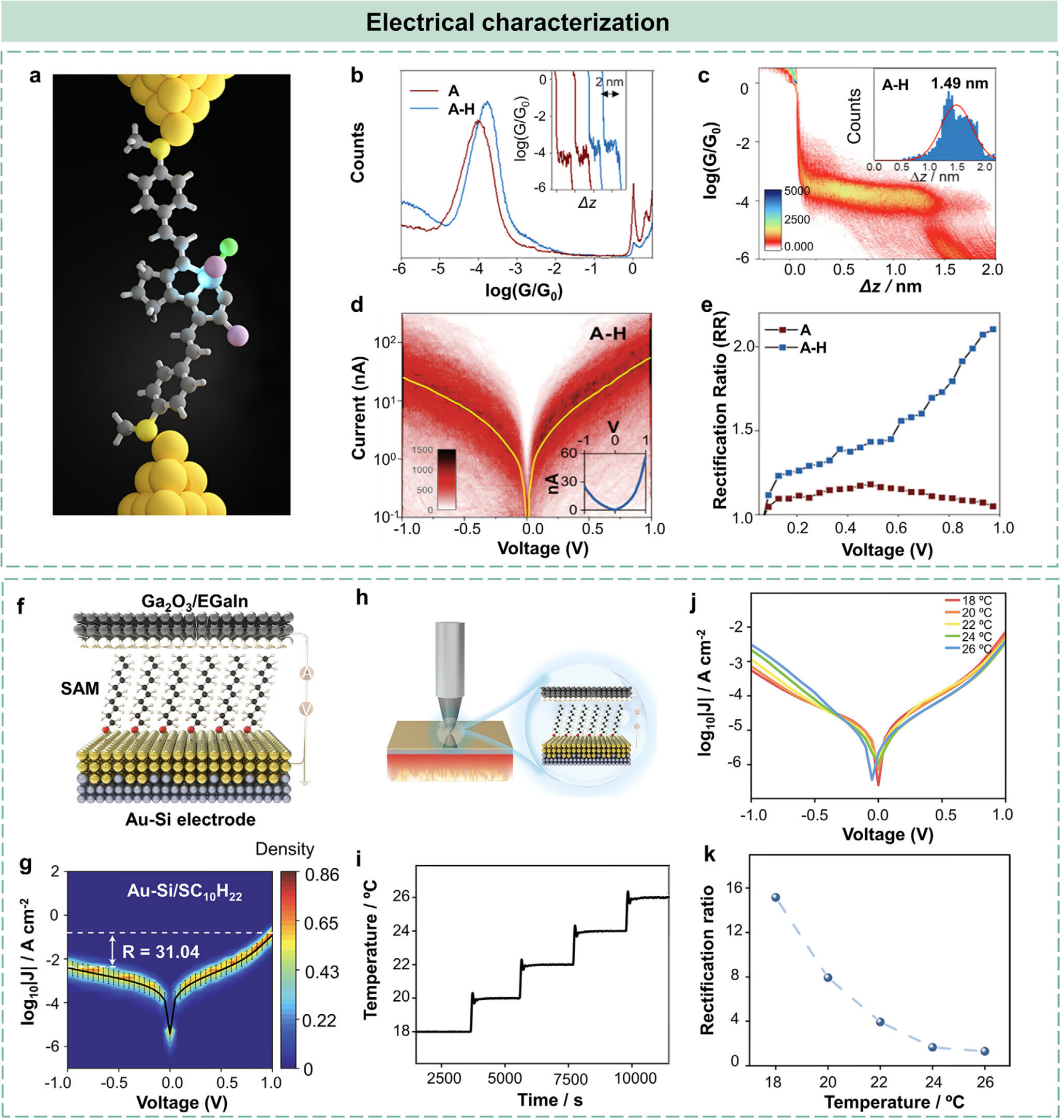
Electrical characterization. (a) Molecular structures and device architecture. (b) One‐dimensional (1D) conductance histograms and typical traces for A (brown) and A‐H (blue). (c) Two‐dimensional (2D) conductance histograms of A‐H with corresponding distributions of stretching distances shown in the insets. (d) 2D histograms of the *I*–*V* characteristics for A‐H. (e) Variation of the RR as a function of the applied voltage. Reproduced with permission from Ref. [185]. Copyright 2023, American Chemical Society. (f) Schematic of the Au–Si/SAM//Ga_2_O_3_/EGaIn architecture. (g) Heat maps of the semilog_10_|*J*| as a function of *V* for the molecular junction. (h) Schematic diagram of the experimental setup for temperature‐dependent measurements. (i) Real‐time temperature curves. (j) Log_10_|*J*| curves plotted as a function of *V* for junctions measured at 18, 20, 22, 24, and 26 °C. (k) RR plotted as a function of temperature. Reproduced with permission from Ref. [115]. Copyright 2024, American Chemical Society.

In contrast, static junctions generally involve direct *I–V* measurement after device fabrication (Figure 15f). Fixed electrode spacing minimizes selection bias associated with stretching trajectories, enabling each measurement to reflect device characteristics. However, as the specific molecular configuration is uncontrolled, measurements may only represent certain junctions. Consequently, repeated measurements across multiple devices, combined with Gaussian fitting or similar statistical analysis, are required to obtain representative *I–V* or current density–voltage (*J–V*) curves (Figure 15g) [235, 236].

Recently, electrically driven non‐equilibrium measurements have attracted increasing attention in the electrical characterization of single‐molecule devices, including transient current measurements based on pulsed bias and all‐electronic pump–probe techniques [237, 238]. Pulsed bias can be used to drive molecular junctions into non‐equilibrium states, while transient current measurements capture their time‐dependent responses. All‐electronic pump–probe methods further enable the evolution of transient excited states and their relaxation dynamics to be tracked by varying the delay time between the pump and probe pulses [239]. In addition, electrically controlled non‐equilibrium interferometric measurements can provide access to electron phase information during single‐molecule transport [240]. Compared with steady–state *I–V* characterization, these approaches provide time‐resolved or phase‐sensitive insights into molecular transport dynamics, offering new opportunities to investigate transient molecular states and non‐equilibrium charge transport processes. Temperature‐dependent conductance measurements are another commonly used method for elucidating transport mechanisms (Figure 15h) [115, 241]. By employing real‐time temperature control to ensure precise thermal stability (Figure 15i), *I–V* curves can be recorded across a specified temperature range (Figure 15j). This approach allows differentiation between thermally activated tunneling, coherent tunneling, and multistep hopping, providing parameters for theoretical modeling. Evaluating RR as a function of temperature also reveals the temperature dependence of rectification (Figure 15k). Device stability and reliability should also be evaluated [242, 243]. Repeated *I–V* cycling and prolonged bias tests assess junction durability and current drift. For instance, EGaIn‐based molecular junctions can sustain hundreds to thousands of repeated scans with stable output, supporting their use in molecular rectification studies [244]. Single‐molecule break junctions instead allow statistical analysis of repeated junction‐formation events, through which molecular transport properties can be evaluated.

In summary, dynamic break junctions and static devices provide complementary electrical measurements. Dynamic junctions allow statistical analysis of repeated junction‐formation events, whereas static devices are suitable for evaluating integrated device characteristics. Together, these measurements are used to examine nonlinear charge transport and rectification in molecular junctions.

### 5.3 Electronic Structure Characterization

The rectification behavior of molecular junctions depends on the relative alignment between MO energies and the Fermi level of the electrodes. Accordingly, electronic structure characterization is commonly used in the study of molecular rectifiers. Determination of the HOMO–LUMO gap, orbital ordering, and their evolution under device operating conditions provides information on charge transport mechanisms and informs molecular design, interface engineering, and device optimization.

Conventional methods, including ultraviolet–visible absorption spectroscopy (UV–vis), ultraviolet photoelectron spectroscopy (UPS), and cyclic voltammetry (CV), are commonly combined to characterize molecular electronic structures [245, 246, 247]. The characterization of a boron‐doped single‐molecule vdW diode illustrates the combined use of these techniques (Figure 16a). UV–vis spectroscopy measures the absorption of photons in the UV and visible ranges, providing optical gap information [55]. As shown in Figure 16b, the UV–vis absorption spectra of solutions and neat films can reveal the impact of molecular packing on electronic transitions. Peak positions often reflect the energy difference between HOMO and LUMO levels, while peak intensities correlate with chemical bond types and functional groups, offering additional insight into conjugation and molecular architecture. However, UV–vis probes the ensemble average in solution or film, but does not directly resolve the local environment within solid‐state molecular junctions. UPS, in contrast, can be used to determine the HOMO level of molecules and the work function of electrodes, enabling evaluation of interfacial energy‐level alignment and the presence of Fermi‐level pinning. The secondary electron cutoff can be used to extract the work function of the system (Figure 16c), while the valence band spectrum can be used to determine the HOMO onset energy, reflecting the energy‐level alignment of the system (Figure 16d). By combining UPS‐determined HOMO energies with the optical gaps from UV–vis, the LUMO level can be indirectly estimated, providing a practical reference for understanding molecule–electrode energy alignment. In addition, CV offers a method for estimating molecular energy levels from redox potentials (Figure 16b). Yet, these measurements are sensitive to the reference electrode and solution environment, and thus should be interpreted primarily as solution‐phase energy estimates rather than direct analogues of solid‐state junction alignment. Fluorescence spectroscopy can further characterize the radiative relaxation behavior of molecular excited states, helping to corroborate the electronic transition features reflected by UV–vis spectroscopy and providing complementary information on excited‐state structures and conjugation. These methods provide complementary information on molecular electronic structure but remain limited in resolving single‐molecule variations and detailed interfacial electronic states.

**FIGURE 16:**
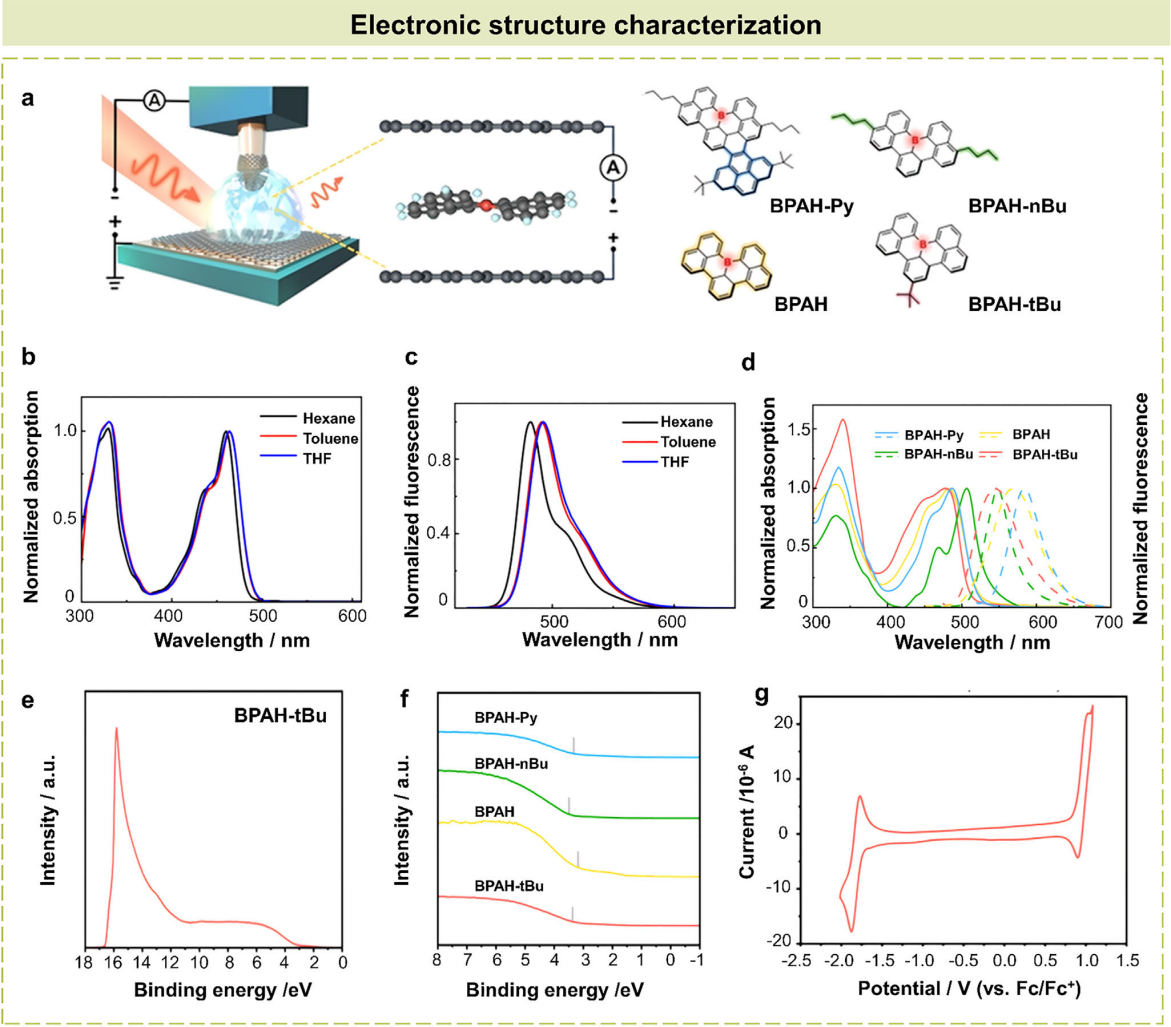
Electronic structure characterization. (a) Schematic diagram of the boron‐doped single‐molecule van der Waals diode alongside the chemical structures utilized as functional building blocks. (b) Cyclic voltammetry (CV, red), ultraviolet–visible (UV–vis) absorption, and fluorescence spectra (green) of tert‐butyl‐substituted boron‐doped polycyclic aromatic hydrocarbon (BPAH‐tBu). (c) Photoemission cutoff spectra of BPAHs single‐molecule two‐dimensional van der Waals heterojunctions (M‐2D‐vdWHs). The black lines indicate the secondary electron cutoff. (d) Valence band spectra of BPAHs M‐2D‐vdWHs after the XPBJ operation. The gray lines indicate the HOMO onset energies.Reproduced with permission from Ref. [55]. Copyright 2025, Wiley‐VCH GmbH.

To address these limitations, transport‐based characterization techniques have been increasingly adopted in molecular rectifier studies. Transition voltage spectroscopy (TVS) analyzes the *I–V* characteristics to extract the bias voltage associated with the dominant MO, which serves as an approximate measure of the tunneling barrier height [248, 249]. As illustrated in the study of Pyr‐Cn SAM junctions (Figure 17a), the minimum in the Fowler–Nordheim (F–N) plot (Figure 17b) is commonly used to define the transition voltage (V_T_) and can provide useful information for analyzing junction nonlinearity and the position of molecular energy levels relative to the Fermi level. However, this minimum alone should not be taken as direct evidence of a physical transition between different transport mechanisms, and its interpretation depends on the specific transport model and device conditions [250]. Specifically, the lower V_T_ values observed for Pyr‐C4S2 compared to Pyr‐C12 (Figure 17c) reveal that the tunneling barrier is highly sensitive to the molecular orientation and packing density of the pyrenyl moieties rather than just the chemical nature of the alkyl chains. Inelastic electron tunneling spectroscopy (IETS) further reveals vibrational contributions during electron tunneling, providing information that can help distinguish transport processes such as resonant tunneling, off‐resonant tunneling, or multistep hopping [179]. For a diblock molecular diode (Figure 17d), while the inherent conductance asymmetry can obscure standard features in the *G*–*V* curve, clear antisymmetric peaks emerge in the IET spectra (Figure 17e). The observation that phonon modes occur at identical energies for both bias polarities, despite differences in current magnitude demonstrates that the potential profile does not shift the vibrational excitation levels, supporting tunneling as the dominant transport mechanism (Figure 17f). Kelvin probe force microscopy (KPFM) offers nanoscale mapping of local contact potential differences, providing indirect information on interfacial dipoles, local charge rearrangements, and molecular orientation, all of which modulate rectification behavior [113]. Combining conductive‐probe AFM (CP‐AFM) with KPFM (Figure 17g) enables the spatial mapping of surface potentials (Figure 17h,i) and shows how the work function of the bottom electrode can be tuned by the molecular length (Figure 17j). Such quantitative data on interfacial energy‐level alignment are useful for examining the electrostatic environment associated with rectification. Compared with conventional optical or electrochemical approaches, TVS, IETS, and KPFM provide transport‐related quantitative or spatially resolved data for investigating non‐equilibrium states and local heterogeneity.

**FIGURE 17:**
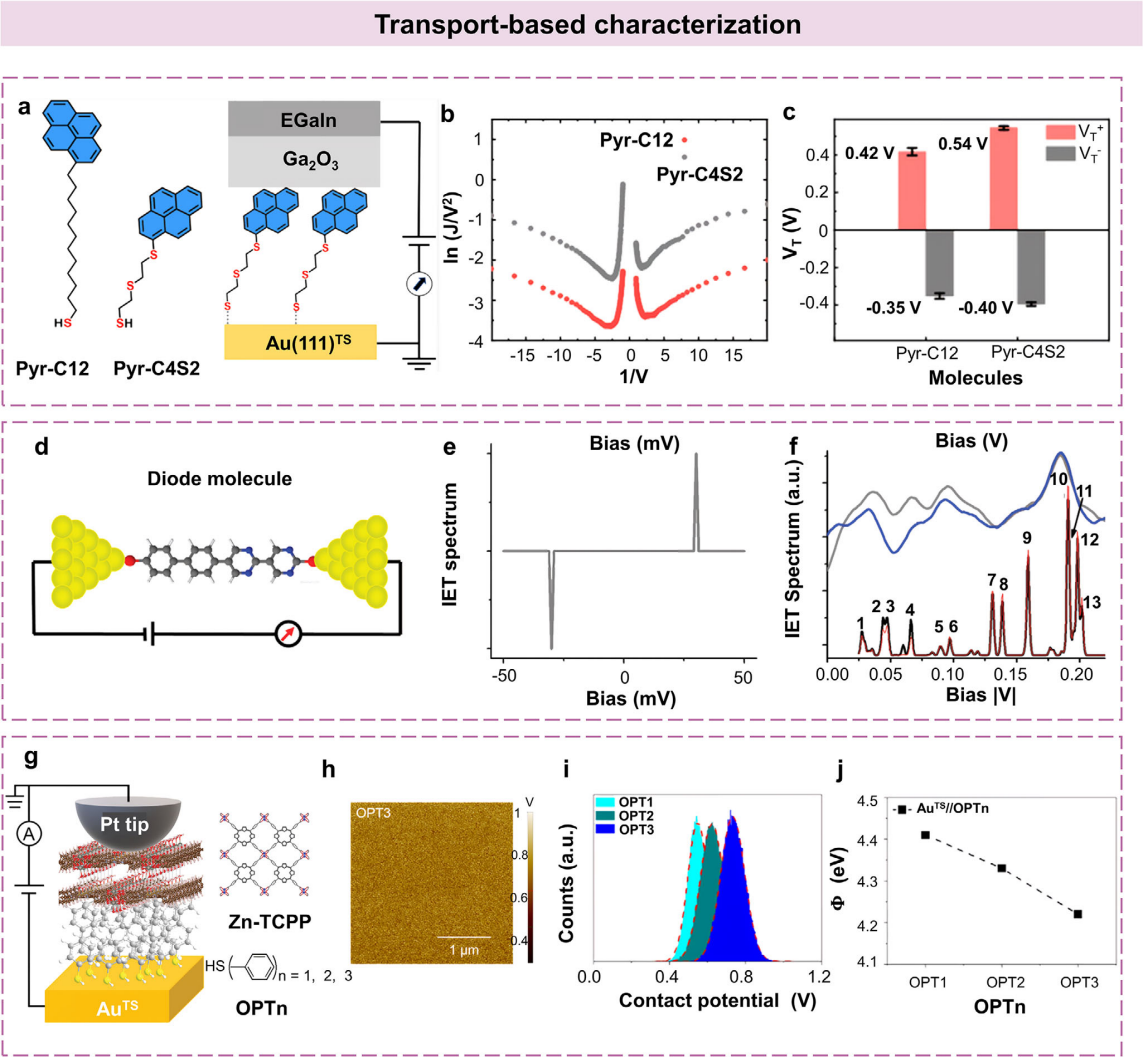
Transport‐based characterization. (a) Schematic illustration of a two‐terminal SAM junction and the chemical structures of Pyr‐C12 and Pyr‐C4S2. (b) Fowler–Nordheim (F–N) plots for Pyr‐C12 and Pyr‐C4S2 SAMs. (c) Derived transition voltages at positive V_T_^+^ and negative V_T_^−^ biases for the corresponding molecular junctions. Reproduced with permission from Ref. [250]. Copyright 2023, American Chemical Society. (d) Depiction of the diblock molecule designed to behave as a molecular diode via its asymmetric electronic structure. (e) Antisymmetric peaks obtained by inelastic electron tunneling spectroscopy (IETS), indicating molecular vibrational signatures. (f) Subtracted IET spectra obtained by taking the derivative of the IET signal and comparing the experimental curves at positive (blue) and negative (gray) bias with calculated spectra (black and red). Reproduced with permission from Ref. [179]. Copyright 2011, American Chemical Society. (g) Schematic of the electronic measurement setup for molecular heterojunctions using CP‐AFM with 2D Zn‐TCPP nanosheets on OPTn SAMs. (h) KPFM images of OPT3 SAMs on Au^TS^ substrates. (i) Histograms of the contact potential for OPTn (*n* = 1, 2, 3) SAMs. (j) Correlation between the work function (Φ) and the contact potential across the OPTn series with varying molecular lengths. Reproduced with permission from Ref. [113]. Copyright 2025, Wiley‐VCH GmbH.

Nevertheless, current electronic structure characterization approaches remain constrained. UV–vis, UPS, and CV are typically performed under idealized solution or vacuum conditions, while IETS and KPFM measurements are conducted under environments that differ from actual device operation, limiting their ability to capture single‐molecule variability and realistic interfacial behavior. Moreover, interpretation of IETS and KPFM data often depends on theoretical modeling. Future work could focus on in situ, multimodal approaches that couple electronic structure characterization with simultaneous electrical transport measurements, allowing molecular junctions to be monitored under operating conditions [53, 251]. Such approaches may help relate measurements obtained under idealized conditions to junction behavior during device operation and clarify the electronic structure and transport processes associated with molecular rectification.

## 6 Theoretical Modeling and Simulation

First‑principles simulations are built upon the fundamental laws of quantum and statistical mechanics and require no empirical parameters. These methods allow calculations of electronic structures and rectification behavior and can be used to investigate the mechanisms underlying *I–V* responses. A widely used theoretical framework builds on the combination of DFT and NEGF methods [37, 252]. This section highlights the key computational steps and applications of this framework in molecular rectifier research. Electronic structure inputs are first established using DFT, followed by NEGF‐based simulations of quantum transport under non‐equilibrium conditions, thereby linking molecular‐level electronic structure with device‐level transport.

Reliable electronic‐structure calculations are important for molecular transport simulations. DFT, as a widely used method for molecular‐scale electronic structure calculations, uses electron density rather than many‐electron wavefunctions as its fundamental variable [252, 253, 254, 255]. This formulation generally reduces the computational cost compared with explicit many‐electron wavefunction methods. Within the Born–Oppenheimer approximation, electronic and nuclear motions are separated, allowing the electronic structure to be calculated at fixed nuclear positions. Kohn–Sham DFT represents the interacting many‐electron system using an auxiliary noninteracting system in an effective potential. Solving the Kohn–Sham equations yields the ground‐state electron density and total energy [256]. Owing to its balance of accuracy and efficiency, DFT has become an important theoretical tool in the study of molecular rectifiers.

In molecular rectifier studies, the most common application of DFT is the calculation of intrinsic molecular electronic structures [100, 140]. By determining the energy levels and spatial distributions of the HOMO and the LUMO, DFT can provide information on the electronic factors contributing to rectification (Figure 18a) [257, 258]. Such calculations are useful for analyzing D–A type rectifying systems, whose rectification is associated with the energy splitting and asymmetric spatial distribution of frontier orbitals, which can be qualitatively predicted by DFT calculations. In addition, key physical quantities such as dipole moments and polarizabilities can be computed, providing insight into molecular responses under external electric fields, which are associated with asymmetric rectification [96]. Moreover, charge density difference analysis and Bader charge decomposition enable quantitative evaluation of molecule–electrode charge transfer and interface dipole formation, clarifying the role of MIGS in level alignment and transport performance [99, 259, 260].

**FIGURE 18:**
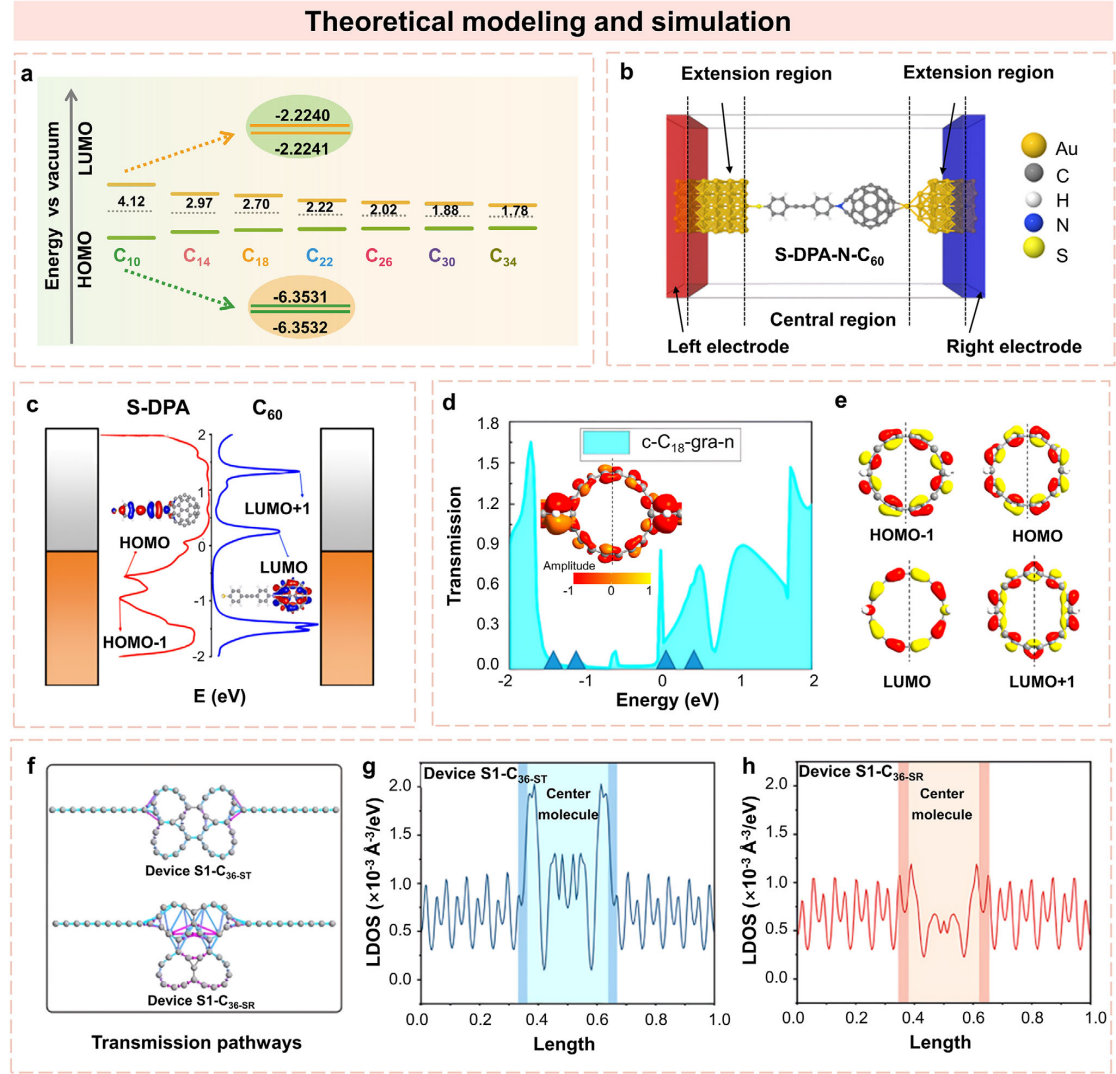
Density functional theory (DFT) and non‐equilibrium Green's function (NEGF) calculations. (a) FMO energies of cyclo[*n*]carbon. The Fermi level is indicated by the gray dashed line. Reproduced with permission from Ref. [257]. Copyright 2025, Royal Society of Chemistry. (b) Schematic representation of the S‐diphenylacetylene (DPA)‐N‐C_60_ device model, where the red and blue boxes denote the electrode sections. (c) Real‐space local density of states (RLDOS) for the S‐DPA (left red line) and C_60_ (right blue line) moieties at 0 V, with the inset illustrating the HOMO and LUMO isosurfaces. Adapted with permission from Ref. [127]. Copyright 2025, Royal Society of Chemistry. (d) Equilibrium transmission spectrum of the c‐C_18_‐gra‐n device. Four molecular projected self‐consistent Hamiltonian (MPSH) eigenvalues near the Fermi level are marked by blue triangles, and the Fermi level is uniformly set to 0 eV. The inset displays the transmission eigenstate corresponding to the main transmission peak of the device under equilibrium conditions. (e) MPSH states corresponding to four frontier molecular orbitals. Reproduced with permission from Ref. [282]. Copyright 2020, American Chemical Society. (f) Transmission pathways of device S1‐C_36‐ST_ (upper) and device S1‐C_36‐SR_ (lower) under equilibrium conditions. (g–h) Local density of states (LDOS) for device S1‐C_36‐ST_ (g) and device S1‐C_36‐SR_ (h). Reproduced with permission from Ref. [283]. Copyright 2025, AIP Publishing.

The choice of exchange–correlation functional affects the reliability of the calculated results. The generalized gradient approximation (GGA, e.g., PBE) remains widely used due to its robustness in structural and band calculations, but systematically underestimates gaps and level alignments [261, 262]. Hybrid functionals (e.g., B3LYP, HSE06), by incorporating a fraction of Hartree‐Fock exchange, improve band‐gap predictions, while long‐range corrected functionals (e.g., ωB97X‐D, CAM‐B3LYP) perform better in describing charge transfer and polarization effects [263, 264, 265, 266, 267]. For systems containing partially occupied and relatively localized *d* or *f* states, DFT+U can be used to reduce the excessive delocalization of localized electronic states in conventional DFT, and its applicability depends on the degree of orbital localization and the specific electronic structure of the system [268]. When the correlated orbitals are strongly hybridized and highly delocalized, the correlated subspace and Hubbard *U* parameter should be chosen carefully. In such cases, hybrid functionals, GW methods, or other electronic‐structure approaches may be more appropriate. Time‐dependent DFT (TD‐DFT), meanwhile, can be used to describe excited states and dynamical processes [269]. Commonly used computational packages include Gaussian 16 for calculating molecular energy levels and spectra; VASP (Vienna Ab initio Simulation Package) for interface modeling; QuantumATK for integrated electronic‐structure and transport calculations; and SIESTA and TranSIESTA for calculations of large systems [270, 271, 272, 273].

Despite its broad use, DFT has several limitations. Kohn‐Sham eigenstates are mathematical constructs rather than true physical observables, leading to systematic deviations of up to ∼1 eV in predicting ionization potentials (IP) and electron affinities (EA) compared with experiments or higher‐level theories such as GW [274]. This uncertainty impacts the alignment between molecular energy levels and electrode Fermi energies, which in turn influences tunneling resonances, current magnitude, and RRs. More accurate methods such as GW or coupled‐cluster theory (CCSD) can improve predictions for relevant electronic properties but remain computationally demanding for realistic device scales [275, 276]. Emerging strategies, including machine‐learning‐assisted functional optimization, high‐throughput screening, and GPU acceleration, are beginning to alleviate the accuracy–efficiency trade‐off [277]. Meanwhile, multiscale quantum mechanical/molecular mechanical (QM/MM) coupling schemes provide a pathway toward realistic large‐scale simulations [278]. These developments extend electronic‐structure calculations toward larger and more complex device models and may support the computational design of molecular rectifiers.

In contrast to DFT, which primarily focuses on molecular and interface electronic structures, the DFT–NEGF framework provides a framework for studying non‐equilibrium transport in molecular rectifiers under bias [279]. By combining DFT electronic structures with NEGF, researchers can compute *I–V* characteristics from first principles and evaluate RRs [280]. This approach allows microscopic transport mechanisms to be compared with experimental measurements.

In device modeling, the system is typically partitioned into a left electrode, a central scattering region, and a right electrode [281]. As illustrated by the S‐diphenylacetylene (DPA)‐N‐C_60_ device model (Figure 18b), the central region includes not only the molecule of interest but also several electrode atoms, forming an “extended molecule” [127]. This treatment accounts for hybridization between MOs and electrode states, a key factor in ensuring physical reliability [131]. The computational procedure typically begins with self‐consistent DFT calculations to obtain the Hamiltonian and electronic states of the central region. Within the NEGF framework, the electrode self‐energy matrices are then introduced to simulate semi‐infinite electrodes. This approach allows for the calculation of *I–V* characteristics and the RR. In transport analysis, the real‐space local density of states (RLDOS) at zero bias and the corresponding HOMO and LUMO isosurfaces can reveal the equilibrium electronic distribution characteristics [127]. The spatial asymmetry of the orbital distribution can may provide a preliminary indication of possible rectification behavior (Figure 18c). To further analyze the microscopic transport mechanism, the evolution of the transmission spectrum T(*E*, *V*) as a function of energy and bias can be calculated to determine the alignment of MOs with the electrode Fermi level. The profile of the transmission spectrum reflects the resonant coupling between molecules and electrodes (Figure 18d) [282]. The molecular orbit dominating charge transport is not always the intrinsic orbital closest to the Fermi level. A combined analysis of the transmission eigenstates of the device and the molecular projected self‐consistent Hamiltonian (MPSH) can help identify the spatial transport channel that contributes the most to the current. As shown in Figure 18d,e, although the system contains multiple MOs, orbital projection analysis reveals that the LUMO+1 becomes the dominant transport orbital due to its higher spatial overlap with the electrode states. To examine electron transport within the device, transmission pathway analysis can show the distribution and evolution of current along the molecular skeleton and at the interfaces in real space (Figure 18f) [283]. Meanwhile, the coupling strength between molecules and electrodes can be quantitatively evaluated by analyzing the distribution of the local density of states (LDOS) at the interface (Figure 18g,h). A higher interfacial LDOS may indicate stronger orbital hybridization and increased charge injection. The above multidimensional simulation analysis provides information for evaluating the effects of molecular structure and interface engineering on RR. The orbital evolution and transport mechanism under external bias can be visualized, especially the energy shift and spatial redistribution of the orbitals. These analyses can provide information on the origin and bias‐dependent evolution of molecular rectification at the atomic scale.

Furthermore, this methodology enables a systematic assessment of how molecular conformation, anchoring groups, electrode materials, and contact geometries influence rectification performance [95, 173, 259]. The DFT–NEGF framework can capture QI effects and can also be used to examine external‐field‐induced orbital evolution, such as those triggered by gate modulation or mechanical strain [134, 135]. Several computational platforms have implemented the DFT–NEGF approach, including TranSIESTA, QuantumATK, OpenMX, and GPAW, all of which support self‐consistent transport calculations under bias and facilitate detailed modeling of molecule–electrode coupling [272, 284, 285, 286].

DFT‑NEGF thus bridges molecular electronic structures and non‑equilibrium transport calculations and is employed to examine device and interface effects. Yet challenges remain, including high computational cost, convergence difficulties, and insufficient treatment of strong correlation and inelastic scattering. More advanced methods, such as multiconfiguration pair‐density functional theory combined with NEGF (MCPDFT–NEGF), dynamical mean‐field theory (DMFT), and density matrix embedding theory combined with NEGF (DMET–NEGF), could in principle provide a more accurate description of transport properties in strongly correlated molecular systems [287, 288, 289]. However, these approaches have not yet been widely implemented in conventional quantum chemistry packages and remain under methodological development. Future directions will focus on improving computational efficiency for large and multiphysics systems, integrating advanced many‐body Green's function methods, developing models for strongly correlated systems, and leveraging machine learning for transport prediction [290]. These developments may improve transport calculations for larger molecular junctions and support the computational evaluation of molecular rectifier designs.

## 7 Comparison of Representative Molecular Rectifiers

In recent years, molecular rectifiers have witnessed substantial advances in performance optimization, regulation strategies, and device construction. Tables 1A and 1B summarize representative experimentally and theoretically predicted molecular rectification systems, respectively, with comparisons covering device platforms, rectification performance, operating conditions, fabrication approaches, and characterization techniques. Since different molecular rectification platforms differ in the number of active molecules, transport measurement methods, and current normalization schemes, their performance should be evaluated within the context of specific device configurations and experimental conditions rather than solely based on the reported RRs.

**TABLE 1A:** Comparison of Representative Experimental Molecular Rectifiers.

Molecule	Device Platform	Electrodes	RR_max_ (Bias)	Measurement Conditions	Modulation Strategies	Assembly/ Fabrication Method	Characterization Techniques	Computational Methods	Refs.
**C_6_‐DNTT**	Ensemble	Pt/Ti	5.0 × 10^8^ (+4.0 V)	Room temperature	Molecular structure Interface	MOC	AFM, CA, J–V, J–V–T, KPFM, SEM, SHG, STEM, UPS, and UV–vis	—	[24]
**HSC_15_Fc–C≡C–Fc**	Ensemble	Pt/EGaIn	6.3 × 10^5^ (−3.0 V)	Ambient	Molecular structure Interface	SAM	AFM, CV, DSC, HRMS, J–V, J–V–T, NEXAFS, NMR, UPS, XPS	MD (NAMD)	[22]
**TCNQ‐doped** **Cu–BTEC**	Ensemble	Au/EGaIn	3.1 × 10^5^ (+5.0 V)	300–450 K	Molecular structure Interface Temperature	SAM	CA, CV, EPR, IRRAS, I–V, I–V–T, UV–vis, XPS, XRD	—	[291]
**HSC_15_Fc–C≡C–Fc**	Ensemble	Pt/EGaIn	1.0 × 10^5^ (−3.0 V)	—	Molecular structure Interface Electric field	SAM	CV, DSC, HRMS, J–V, NEXAFS, NMR, UPS, XPS	MD (Gromacs)	[292]
**T‐exTTF**	Ensemble	Au/EGaIn	5.0 × 10^4^ (−1.5 V)	250–330 K	Molecular structure Interface Temperature	SAM	CV, Ellipsometry, HRMS, J–V, J–V–T, NMR, UPS, XPS, XRD	DFT (Gaussian) DFT–NEGF (QuantumATK)	[195]
**S(CH_2_)_11_MV^2+^I_2_^−^**	Ensemble	Ag/EGaIn	2.5 × 10^4^ (−1.0 V)	—	Molecular structure Interface	SAM	ARXPS, CV, HRMS, J–V, NEXAFS, NMR, UPS, UV–vis, XPS	DFT (Gaussian, VASP)	[189]
**Fc–BPT**	Ensemble	Au/EGaIn	2.0 × 10^3^ (−0.2 V)	Ambient	Molecular structure Interface	SAM	CV, J–V, NEXAFS, TVS, XPS	DFT (Gaussian)	[241]
**HBQ**	Ensemble	Au/EGaIn	178.0 (−2.5 V)	240–330; Vacuum	Molecular structure Interface Temperature	SAM	AFM, CV, J–V, J–V–T, NEXAFS, STM, UPS, UV–vis	DFT (Gaussian) TD‐DFT (Gaussian)	[293]
**SC_11_PYR**	Ensemble	Ag/EGaIn	172.0 (−0.74 V)	98–298 K; Vacuum	Molecular structure Interface	SAM	AFM, CA, HRMS, J–V, J–V–T, NMR, TVS, UPS, UV–vis, XPS	DFT	[140]
**S(CH_2_)_11_S–BTTF**	Ensemble	Au/EGaIn	124.0 (+1.5 V)	170–320 K; Vacuum	Molecular structure Interface Temperature	SAM	ARXPS, CV, J–V, J–V–T, NEXAFS, UPS	—	[149]
**S(CH_2_)_11_BIPY–CoCl_2_**	Ensemble	Au/EGaIn	82.0 (+1.0 V)	200–320 K; Vacuum	Molecular structure Interface Temperature	SAM	CV, J–V, J–V–T, UPS, XPS	DFT (Gaussian)	[114]
**S(CH_2_)_10_OPhBr_5_**	Ensemble	Ag/EGaIn	40.0 (+1.0 V)	250–340 K	Molecular structure Interface Temperature	SAM	AFM, HRMS, J–V, J–V–T, NEXAFS, NMR, UPS, UV–vis, XPS	DFT(Gaussian)	[112]
**S(CH_2_)_11_CO_2_H**	Ensemble	Au/EGaIn	32.0 (+1.5 V)	1%–3% O_2_/N_2_; RH < 10%	Molecular structure Interface Optical Field Chemical Environment	SAM	AFM, J–V, KPFM, XPS	DFT (Gaussian)	[158]
**TTF‐PTM**	Ensemble	Au/EGaIn	13.0 (−2.0 V)	—	Molecular structure Interface	SAM	AFM, CV, ESR, FT‐IR, J–V, NEXAFS, NMR, UV–vis, UPS, XPS	DFT(Gaussian, SIESTA) DFT–NEGF (QuantumATK)	[244]
**S(CH_2_)_2_CONR^1^R^2^**	Ensemble	Au/EGaIn	12.4 (+1.0 V)	—	Molecular structure Interface	SAM	ARXPS, CA, HRMS, J–V, NMR, XPS	MD (LAMMPS)	[242]
**Phenyl benzoate**	Single‐molecule	Graphene/ Graphene	6.4 × 10^3^ (+1.0 V)	373 K; AlCl_3_	Quantum interference Electric field Chemical environment	Carbon‐based junction	IETS, I–t, I–V	DFT (Gaussian) DFT–NEGF (QuantumATK)	[46]
**1,8‐Nonadiyne**	Single‐molecule	N‐type Si/Au‐Tip	4.0 × 10^3^ (−1.5 V)	Ambient	Molecular structure Interface	STM‐BJ	AFM, I–V, I–t, XPS	DFT (Gaussian)	[182]
**DPE‐2F**	Single‐molecule	Au/Au	620.0 (+1.2 V)	6 K; Vacuum	Molecular structure Electric Field	MCBJ	I–V, SEM	DFT–NEGF (ADF)	[141]
**BPAH–tBu**	Single‐molecule	Graphene/ Graphene	457.0 (+1.0 V)	Room temperature	Molecular structure Interface	XPBJ	CV, HRMS, I–V, NMR, RS, TVS, UPS, UV–vis, XRD	DFT (Gaussian) DFT–NEGF (QuantumATK) TD‐DFT (Gaussian)	[55]
**Ru‐DAE**	Single‐molecule	Graphene/ Graphene	61.0 (±1.0 V)	Room temperature; Vacuum	Electric Field Optical Field	Carbon‐based	HRMS, I–V, I–t, NMR, SEM, UV–vis	DFT (Gaussian, VASP) DFT–NEGF (Nanodcal)	[26]
**Py/NDI**	Single‐molecule	Au/Au	16.0 (±1.0 V)	Room temperature; Mesitylene	Quantum interference Molecular structure	MCBJ	Flicker Noise, HRMS, I–V, NMR, SEM, UV–vis	DFT(Gaussian) DFT–NEGF (QuantumATK)	[47]
**IT‐6**	Single‐molecule	Au/Au‐Tip	3.6 (+0.5 V)	298 K; NaF	Molecular structure Chemical Environment	STM‐BJ	CV, I–V, NMR, UV–vis	DFT (Gaussian) TD‐DFT (Gaussian)	[25]
**PPCNMe_2__d**	Single‐molecule	Au/Au‐Tip	2.6 (+1.5 V)	Mesitylene	Molecular structure Interface	STM‐BJ	I–V, NMR, PL, TVS, UV–vis	DFT (Gaussian) DFT–NEGF (QuantumATK)	[96]
**Hetero π‐stacked complex 1·(2·3)**	Single‐molecule	Au/Au‐Tip	2.0 (+0.5 V)	Ambient	Molecular structure Interface	STM‐BJ	I–V, NMR, STM	DFT (Gaussian) DFT–NEGF	[101]

**TABLE 1B:** Comparison of Representative Computational Molecular Rectifiers.

Molecule	Device Platform	Electrodes	RR_max_ (Bias)	Modulation Strategies	Computational Methods	Refs.
**HSC_11_BIPY–C≡C–BIPY**	Single‐molecule	Pt/Pt	8.3 × 10^6^ (+1.2 V)	Molecular structure Interface	DFT (QuantumATK) DFT–NEGF (QuantumATK)	[107]
**SC_n_BIPYC_11_**	Single‐molecule	Ag/Ag	422.0 (−1.8 V)	Molecular structure Interface	DFT (QuantumATK) DFT–NEGF (QuantumATK)	[93]
**Pyrene‐(CH)_3_‐** **(CH_2_)_3_‐(CH)_3_‐benzene**	Single‐molecule	Au/Au	93.8 (−0.8 V)	Molecular structure	DFT (VASP) DFT–NEGF (QuantumATK) MD (Virtual NanoLab)	[98]
**C_10_‐C_12_**	Single‐molecule	Graphene nanoribbon/Carbon chain	23.0 (−0.4 V)	Molecular structure Interface	DFT (Gaussian) DFT–NEGF (QuantumATK)	[280]
**N‐Phenylbenzamide**	Single‐molecule	Au/Au	16.7 (+0.2 V)	Molecular structure	DFT(Gaussian) DFT–NEGF (TranSIESTA)	[90]
**Pyrimidinyl‐triphenyl**	Single‐molecule	Au/Au	1.7 (+0.8 V)	Molecular structure	DFT (SIESTA) DFT–NEGF (TranSIESTA)	[91]

Notes:

1.Tables 1a and 1b summarize some of the most representative high‐performance molecular rectifiers reported in the past decade.

2.The RR is defined as:$RR=\frac{\max(\left|I(+V)|,|I(-V)\right|)}{\min(\left|I(+V)|,|I(-V)\right|)}$; The positive and negative signs of the bias voltage indicate the rectification direction.

3. Abbreviations used in the tables are defined in the Abbreviations section.

4.Em dashes (—) indicate information that is not applicable or not reported.

From experimentally reported rectification performance (Table 1A), molecular rectifiers based on different platforms exhibit large differences. Ensemble‐based molecular devices generally achieve relatively high RR. For example, SAM‐based molecular rectifiers (e.g., HSC_15_Fc–C≡C–Fc) and ordered molecular layer devices (e.g., C_6_‐DNTT) have demonstrated high RRs, with some systems reaching 10^5^–10^8^ [22, 24]. However, such high RR values are typically obtained from large‐area molecular assemblies, where rectification behavior is influenced not only by molecular structures and interfacial properties but also by molecular packing, device configurations, measurement environments, and operating bias conditions. Therefore, the maximum RRs reported for different systems should be interpreted in the context of their corresponding experimental conditions. In contrast, single‐molecule junctions exhibit a relatively broad distribution of RRs. Their significance extends beyond achieving high RRs, as they enable investigation of molecular structures, molecule–electrode coupling, and quantum transport processes at the single‐molecule level, providing insights into the microscopic origin of rectification. Since theoretical calculations mainly focus on molecular‐scale electronic structures and transport processes, most theoretical systems summarized in Table 1B are based on single‐molecule junction models. These theoretical results provide valuable insights into the relationship between molecular structures and transport behaviors and help identify potential design principles and physical mechanisms. Nevertheless, they are generally obtained under idealized device models and computational conditions and therefore cannot be equated with experimentally achievable device performance.

From the perspective of modulation strategies, rectification behavior in practical molecular devices is usually governed by multiple factors rather than a single regulation mechanism. Molecular structural design and molecule–electrode interface engineering remain the two primary approaches for improving rectification performance. Molecular design mainly regulates intrinsic molecular structures and electronic properties to tune transport asymmetry within the molecule, including the design of molecular backbones, main‐chain structures, donor–acceptor units, and substituents. In comparison, molecule–electrode interface engineering involves the regulation of anchoring groups, electrode materials, and interfacial coupling, which directly affects charge transport between MOs and electrodes. Due to the limitations imposed by practical device fabrication, perfectly symmetric and idealized molecule–electrode interfaces are difficult to achieve. Consequently, interfacial structures and coupling configurations often introduce additional transport asymmetry. In recent years, QI effects and external stimuli, including electric fields, optical, temperature, and chemical environments, have also attracted increasing attention, offering additional approaches for controlling transport pathways and developing functional molecular devices.

Regarding device fabrication and characterization, different molecular rectification platforms rely on different techniques. Molecular‐ensemble devices are commonly fabricated using SAMs, molecular multilayers, and MOCs, followed by static electrical transport measurements for rectification evaluation. In contrast, dynamic single‐molecule junctions typically rely on MCBJ, STM‐BJ, and XPBJ techniques, where repeated junction formation and rupture enable transport measurements at the single‐molecule level. Static single‐molecule junctions are generally constructed using stable nanogap electrode configurations for transport characterization. To analyze device structures, electronic properties, and transport behaviors, molecular rectification studies commonly combine multiple characterization techniques, including structural characterization (NMR, XRD, and AFM), electronic structure analysis (XPS, UPS, and NEXAFS), and electrical measurements (I–V, J–V–T, and TVS). These techniques are used to verify device structures, analyze electronic properties, and evaluate transport characteristics, providing experimental support for understanding rectification performance and transport mechanisms. Meanwhile, theoretical simulations based on DFT, DFT–NEGF, and molecular dynamics (MD) further contribute to the analysis of electronic structures, energy‐level alignment, charge transport, and structural responses under external stimuli, facilitating the interpretation of experimentally observed transport behaviors.

Overall, recent studies on molecular rectifiers have achieved substantial progress in performance optimization, modulation strategies, device fabrication and characterization, as well as theoretical modeling. These developments have established a research framework spanning molecular design, interfacial engineering, and device realization. However, significant challenges remain in translating molecular rectifiers from experimental demonstrations toward practical applications. Single‐molecule systems often face difficulties in simultaneously achieving high RRs and stable device performance. In addition, long‐term stability, structural controllability, and scalable fabrication of molecular devices require further improvement. The integration of molecular rectifiers with existing semiconductor technologies also remains an important challenge for future applications.

## 8 Summary and Outlook

As a promising avenue to surpass the physical limits of conventional silicon‐based technologies in the post‐CMOS era, molecular rectifiers consistently demonstrate unique advantages in ultimate device scaling and potential applications. Despite continuous performance improvements, their practical implementation at the device level still faces considerable challenges. This uncertainty primarily stems from the formidable challenges in realizing molecular devices that combine high RRs, robust stability, and integrability. Fundamentally, these challenges can be distilled into three scientific questions: How can highly controllable and structurally stable devices be constructed experimentally? How can the intrinsic relationship between molecular structure and electronic transport behavior be accurately elucidated theoretically? And how can molecular rectifiers be effectively integrated into complex electronic systems at the application level? This section provides a critical assessment of current bottlenecks across these three dimensions, along with strategies and future perspectives aimed at driving sustained progress in this field.

1. Experimental efforts: constructing robust and controllable molecular rectifiers

The precise fabrication of nanogap electrodes and the controlled assembly of molecule–electrode interfaces remain challenges that limit device performance. As device dimensions approach the single‐molecule scale, rectification characteristics become sensitive to structural fluctuations and interfacial perturbations, placing stringent demands on electronic coupling and chemical stability at the interface. Although break junctions, scanning probe methods, and self‐assembled monolayers have been used to construct molecular junctions, issues such as poor configurational uniformity and limited reproducibility of electrical responses remain common. Moreover, bias‐induced molecular rearrangements, electrode atom migration, and dynamic evolution of interfacial states can cause current fluctuations and device instability during operation. Addressing these challenges will require nanoscale fabrication techniques with improved atomic‐scale control, the use of selective anchoring groups, and the adoption of low‐diffusivity electrode materials to enhance interfacial stability and charge injection efficiency. In addition, the development of in situ multimodal characterization techniques is also important to monitor interfacial dynamics in real time during device operation. Bioinspired materials such as DNA scaffolds and mechanically interlocked molecules, which can exhibit self‐repair capabilities and excellent conductivity, may also offer alternative platforms for building next‐generation, high‐stability molecular devices.

2. Theoretical insights: understanding and predicting rectification mechanisms

Theoretical modeling plays an important role in the design, mechanistic understanding, and performance prediction of molecular rectifiers. While the DFT–NEGF framework has become a widely used modeling framework, its quantitative accuracy in describing energy‐level alignment, MIGS, and interfacial charge redistribution remains limited by the approximations used in the electronic‐structure calculations. In particular, the systematic underestimation of energy gaps by generalized gradient approximation functionals such as PBE can affect the predicted rectification behavior. To enhance predictive accuracy and broaden applicability, it is important to incorporate higher‐level methods such as hybrid functionals, the GW approximation, and TD‐DFT. Multiscale modeling frameworks that combine ab initio calculations with MDs and machine learning approaches may help balance computational efficiency with the complexity of realistic molecular junctions. Developing general transport models that can account for realistic environments, including solvents, electric fields, and mechanical stress, may further improve comparisons between theoretical predictions and experimental measurements and help clarify the transport mechanisms.

3. Toward integration and commercialization

Although experiments have demonstrated high RRs and tunability in molecular rectifiers, their transition toward practical applications is hindered by three main barriers. First, their operational stability and environmental tolerance remain concerns for long‐term operation. Second, current fabrication techniques are not yet compatible with established semiconductor fabrication processes, which limits the scalable and controllable construction of device arrays. Third, the typical output currents of many single‐molecule junctions remain in the picoampere to nanoampere range, which is insufficient to drive conventional electronic circuits, whereas ensemble devices can provide higher currents but face challenges in large‐area current uniformity. One potential pathway toward commercialization involves embedding molecular rectifiers within heterogeneous platforms that integrate CMOS devices, two‐dimensional materials, quantum dots, or flexible electronics. This approach could allow their rectification behavior to be evaluated within integrated electronic systems. Customizing molecular rectifiers for specialized applications such as flexible sensing, biointerfaces, and neuromorphic computing could also offer viable possible application scenarios. Establishing standardized testing protocols, reproducible manufacturing processes, and unified performance evaluation frameworks will be important for comparing results across studies and evaluating their suitability for device integration.

In summary, the transition of molecular rectifiers from scientific prototypes to engineered applications requires not only close integration between theory and experiment but also coordinated advances across disciplines and scales. Despite the significant challenges ahead, the molecular‐scale dimensions and low‐power operation of molecular rectifiers highlight their profound potential in next‐generation nanoelectronic systems. With continued progress in advanced characterization and integrated manufacturing technologies, molecular rectifiers are poised to play an substantive role in future information systems, potentially catalyzing a paradigm shift in microelectronics.

## Author Contributions

**Junnan Guo**: writing – original draft, methodology, conceptualization, investigation, validation. **Shufan Song**: visualization, investigation, methodology. **Wenhui Fang**: methodology, formal analysis. **Jifeng Tang**: validation, investigation. **Wenhao Li**: validation, investigation. **Weikang Wu**: writing – review and editing. **Shishen Yan**: writing – review and editing, resources. **Hui Li**: resources, writing – review and editing, supervision, software. **Lishu Zhang**: writing – review and editing, writing – original draft, resources, supervision.

## Conflicts of Interest

The authors declare no conflicts of interest.

## Data Availability

The data that support the findings of this study are available from the corresponding author upon reasonable request.
